# Disparities and Risks of Sexually Transmissible Infections among Men Who Have Sex with Men in China: A Meta-Analysis and Data Synthesis

**DOI:** 10.1371/journal.pone.0089959

**Published:** 2014-02-24

**Authors:** Eric P. F. Chow, Joseph D. Tucker, Frank Y. Wong, Eric J. Nehl, Yanjie Wang, Xun Zhuang, Lei Zhang

**Affiliations:** 1 The Kirby Institute, University of New South Wales, Sydney, NSW, Australia; 2 Central Clinical School, Faculty of Medicine, Nursing and Health Sciences, Monash University, Melbourne, VIC, Australia; 3 Melbourne Sexual Health Centre, Alfred Hospital, Melbourne, VIC, Australia; 4 Comprehensive AIDS Research Center, School of Medicine, Tsinghua University, Beijing, China; 5 University of North Carolina Project-China, Guangzhou, China; 6 London School of Hygiene and Tropical Medicine, London, United Kingdom; 7 Department of Behavioral Sciences and Health Education, Emory University, Atlanta, Georgia, United States of America; 8 School of Public Health, Nantong University, Nantong, Jiangsu Province, China; Alberta Provincial Laboratory for Public Health/University of Alberta, Canada

## Abstract

**Background:**

Sexually transmitted infections (STIs), including Hepatitis B and C virus, are emerging public health risks in China, especially among men who have sex with men (MSM). This study aims to assess the magnitude and risks of STIs among Chinese MSM.

**Methods:**

Chinese and English peer-reviewed articles were searched in five electronic databases from January 2000 to February 2013. Pooled prevalence estimates for each STI infection were calculated using meta-analysis. Infection risks of STIs in MSM, HIV-positive MSM and male sex workers (MSW) were obtained. This review followed the PRISMA guidelines and was registered in PROSPERO.

**Results:**

Eighty-eight articles (11 in English and 77 in Chinese) investigating 35,203 MSM in 28 provinces were included in this review. The prevalence levels of STIs among MSM were 6.3% (95% CI: 3.5–11.0%) for chlamydia, 1.5% (0.7–2.9%) for genital wart, 1.9% (1.3–2.7%) for gonorrhoea, 8.9% (7.8–10.2%) for hepatitis B (HBV), 1.2% (1.0–1.6%) for hepatitis C (HCV), 66.3% (57.4–74.1%) for human papillomavirus (HPV), 10.6% (6.2–17.6%) for herpes simplex virus (HSV-2) and 4.3% (3.2–5.8%) for *Ureaplasma urealyticum*. HIV-positive MSM have consistently higher odds of all these infections than the broader MSM population. As a subgroup of MSM, MSW were 2.5 (1.4–4.7), 5.7 (2.7–12.3), and 2.2 (1.4–3.7) times more likely to be infected with chlamydia, gonorrhoea and HCV than the broader MSM population, respectively.

**Conclusion:**

Prevalence levels of STIs among MSW were significantly higher than the broader MSM population. Co-infection of HIV and STIs were prevalent among Chinese MSM. Integration of HIV and STIs healthcare and surveillance systems is essential in providing effective HIV/STIs preventive measures and treatments.

**Trial Registration:**

PROSPERO No: CRD42013003721

## Introduction

Men who have sex with men (MSM) is a high-risk population for HIV and sexually transmitted infections (STIs) in China and internationally [1]–[6]. Although MSM only accounts for 2–4% of the total Chinese sexually-active male population [7], nearly a third of new HIV infections were attributable to homosexual contact in 2011 [8]. Statistics showed that the national HIV prevalence among Chinese MSM increased rapidly from 1.2% in 2001 to 6.3% in 2011 [8], [9], and its transmission has been substantially facilitated by the co-existing STI epidemics [10]. For instance, syphilis prevalence among Chinese MSM increased from 6.8% to 13.5% during 2003–2008 [11] and prevalence of HIV/syphilis co-infection doubled from 1.4% in 2005–2006 to 2.7% in 2007–2008 [11]. MSM commonly practice anal and oral sex [12], but other sexual practices such as anilingus, fisting and rimming are prevalent [12], [13]. The diverse types of sexual intercourse and low rate of condom use elevated the risks of STIs transmission [14]–[17]. Also, social stigma and internalised homophobia have led to common psychological disorders, substance abuse and unintended high-risk behaviours [18], [19], which fuel the wide spread of HIV/STIs syndemics [19]–[21].

Co-infection of STI and hepatitis is a major risk for HIV transmission. Prevalence of human papillomavirus (HPV) is extremely high among MSM worldwide (63.9% in HIV-negative and 92.6% in HIV-positive MSM) [22], and the presence of HPV-related high-grade squamous intraepithelial lesions can destroy anogenital mucosal tissues and facilitates the intracellular transmission of HIV [23]. MSM infected with any of herpes simplex virus type 2 (HSV-2), rectal chlamydia, gonorrhoea and syphilis are associated with 3–8-folds higher risk of HIV acquisition [24]–[27]. Co-infection with viral hepatitis enhances the progression of liver diseases; significantly increasing the risk of morbidity and mortality among people living with HIV [28]. Patients with dual HIV/HBV or HIV/HCV infection have a nineteen and three times higher risk of liver death than mono-infected patients, respectively [29]–[31].

Epidemics of STIs and hepatitis infections are largely under-reported among Chinese MSM [32]. Apart from HIV, only syphilis and gonorrhoea are notifiable by law in China [33]. Since the current STIs surveillance in China mainly relies on the passive hospital-based reporting system [34], [35], it is estimated that the reported cases of STIs in China only account for 10% of the actual number of infections [36], [37]. Numerous scattered studies reported prevalence estimates for STIs and hepatitis infections among Chinese MSM, but none provided national estimates for these infections. A comprehensive data synthesis on these prevalence estimates is valuable in providing an overview of the extent of STIs and hepatitis infections disease burden and risk of infections among Chinese MSM. Building on findings of previous studies [9], [11], this study aims to assess the current disease burdens and risks of STIs and hepatitis infections among the broader MSM, HIV-infected MSM and male sex workers (MSW) in China.

## Methods

### Systematic Review and Meta-analysis

This review was conducted in accordance with the PRISMA (Preferred Reporting Items for Systematic Reviews and Meta-Analyses) Statement issued in 2009 (Checklist S1) [38]. The protocol for this review has been prospectively registered (CRD42013003721) with International Prospective Register of Systematic Reviews (PROSPERO) [39].

### Search Strategy

We searched PubMed, Embase, Wanfang Data, VIP Chinese Journal Database (VIP) and China National Knowledge Infrastructure (CNKI) for studies reported the prevalence of STIs and hepatitis infections among MSM in China from January 2000 to February 2013. The search included Medical Subject Headings (MeSH) terms for ‘China’, ‘Chinese’, ‘hepatitis’, ‘sexually transmitted diseases’ and ‘sexually transmitted infections’, and other keywords associated with each STI: ‘chlamydia’, ‘*Chlamydia trachomatis*’, ‘gonorrhoea’, ‘*Neisseria gonorrhoeae*’, ‘genital wart’, ‘hepatitis’, ‘HBV’, ‘hepatitis B’, ‘HCV’, ‘hepatitis C’, ‘HSV’, ‘herpes simplex virus’, ‘HPV’, ‘human papillomavirus’ and ‘*Ureaplasma urealyticum*’. Truncation and wildcard operators were used in the search strategy. Hand searching from the reference lists of the retrieved articles in the above databases was also included. Our search strategy was limited to English and Chinese language. Two reviewers (EPFC, YW) independently screened all retrieved abstracts from the five aforementioned databases against the selection criteria. Discrepancies on which articles met the inclusion criteria were resolved by a third reviewer (LZ).

### Selection Criteria

#### Type of studies

We preferentially looked for quantitative epidemiological studies, including cohort, before-and-after, and cross-sectional studies. Editorials, newspapers, review articles, conference abstracts, modelling studies and case reports were excluded. We only included studies conducted in mainland China. Studies conducted in Hong Kong, Macau and Taiwan were excluded.

#### Type of participants

We only included studies with MSM who self-reported having any homosexual intercourse in the past 12 months. There were no restrictions on age, marital status, educational level, ethnicity, residency and self-identified sexual orientation of the participants. Male sex workers (MSW) are a subpopulation in the Chinese MSM population who commercially sell sex to MSM (also known as ‘money boys’ in China), we only included studies in which MSW constituted <20% of the study sample. Studies targeted HIV-positive MSM were included to investigate the prevalence of co-infection. Studies with sample size of the MSM <30 and HIV-positive MSM population <10 were excluded in this review.

#### Type of outcome measures

We included studies measured the prevalence of the most common STIs among MSM (including chlamydia, genital wart [*Condyloma acuminatum*], gonorrhoea, HPV, HSV-2 and *Ureaplasma urealyticum*). We also included the two rampant and sexually transmissible hepatitis infections (i.e. HBV and HCV). Studies were included if the infection was diagnosed and confirmed by a molecular biology-based method. HBV infection was defined by the presence of hepatitis B surface antigen (HBsAg) as HBsAg is a serologic marker represents either acute or chronic HBV infections rather than due to other cases such as HBV immunity. HCV infection was diagnosed by the presence of anti-HCV antibodies. Enzyme immunoassay (EIA), polymerase chain reaction (PCR) or recombinant immunoblot assay (RIBA) was used to detect HCV. HPV infection was tested by PCR. Chlamydia, genital wart, gonorrhoea and *Ureaplasma urealyticum* were tested by PCR, EIA or cell culture. HSV-2 was diagnosed by the presence of Immunoglobulin G (IgG) by EIA. Self-reported infections were excluded from this review.

### Quality Assessment

The quality assessment of eligible studies was measured according to the checklist tools for assessing quality in observational studies [40]. Six domains were used to assess the risk of bias: (1) methods for selecting study participants; (2) methods for measuring exposure and outcome variables; (3) design-specific source of bias; (4) method of control confounding; (5) statistical methods; and (6) other biases (including conflict of interest and disclosure of funding sources). The quality of each item was categorized as either ‘Low risk (+)’, ‘High risk (−)’ or ‘Unclear (?)’ in accordance with the guideline recommended by the Cochrane Collaboration [41].

### Data Extraction and Management

Data were extracted and entered into a predesigned electronic data collection form in Microsoft Access database (Version 2010, Microsoft Corp., Redmond, WA, USA). Each study was given by a unique ID and the data collection form included information on: (1) study design: location, sampling methods/venues, type of study, sample size, and study year; (2) demographic characteristics of the study participants: age, marital status, and educational level; (3) epidemiology of STIs and hepatitis infections: prevalence estimates of STIs, HBV or HCV, method of laboratory diagnosis, and biomarkers examined.

### Definition of Outcomes

The primary outcome measure was the prevalence of STIs and hepatitis infections among Chinese MSM, and it was expressed as a percentage of the number of infections divided by the number of individuals tested for the infection. To estimate the risk of STIs acquisition in MSM, the pooled STIs prevalence estimates among MSM were compared with MSW and the general population. Data on MSW were collected from a recent published meta-analysis study [42]. The prevalence of the general population was estimated among the Chinese adults of reproductive age (i.e. ≥15 years), and data were collected from the latest cross-sectional study across the country.

### Statistical Analysis

Meta-analyses were performed using the Comprehensive Meta-Analysis software (Version 2.2, Biostat, Englewood, NJ, ISA) [43]. The pooled prevalence estimates for each STI were calculated by combining the weighted prevalence for each study. A standard continuity correction of 0.5 was added to the studies with prevalence of zero [44]. The effect rates of pooled estimates and 95% confidence intervals (CI) for each STI were graphically presented in the form of forest plots.

Heterogeneity tests across studies were detected by the chi-squared based Cochran Q-test (*p<*0.10 indicates statistically significant heterogeneity) and *I* ^2^ statistic [45]–[47]. *I* ^2^ was calculated as *I* ^2^ = [(*Q* – degree of freedom)/*Q*] ×100, where *Q* is the Cochran’s statistic. *I* ^2^ values of 25, 50 and 75 representing low, medium and high heterogeneity, respectively [47]. If high and significant heterogeneity (*I* ^2^>75) was detected across studies [47], the random-effect model was used to calculate the summary of pooled prevalence estimates [48], [49]. Otherwise, a fixed-effect model was used when low heterogeneity was observed across studies. Sampling sizes of the study were taken into account in both models. Evidences on HBV and HCV prevalence estimates were further stratified by six administrative Chinese regions and study years to explore the potential factor(s) that contributed to the heterogeneity. The geographical and temporal trends of STIs could not be investigated in this review due to insufficient studies.

The potential presence publication bias was examined by the inspection of funnel plot [50], and the significant of funnel plot asymmetry was statistically tested using the Begg and Mazumdar rank correlation method (*p<*0.05 represents statistically significant publication bias) [51], [52]. Test of publication bias was performed if the meta-analysis consisted of more than 10 studies [53], [54].

We performed additional analyses to compare the STIs prevalence among Chinese MSM with estimated prevalence among adults in the general population and MSW. Odds ratio (OR) and its 95% CI were calculated to measure the risk of STIs acquisition in MSM/MSW.

## Results

### Characteristics of Included Studies

**Figure 1:**
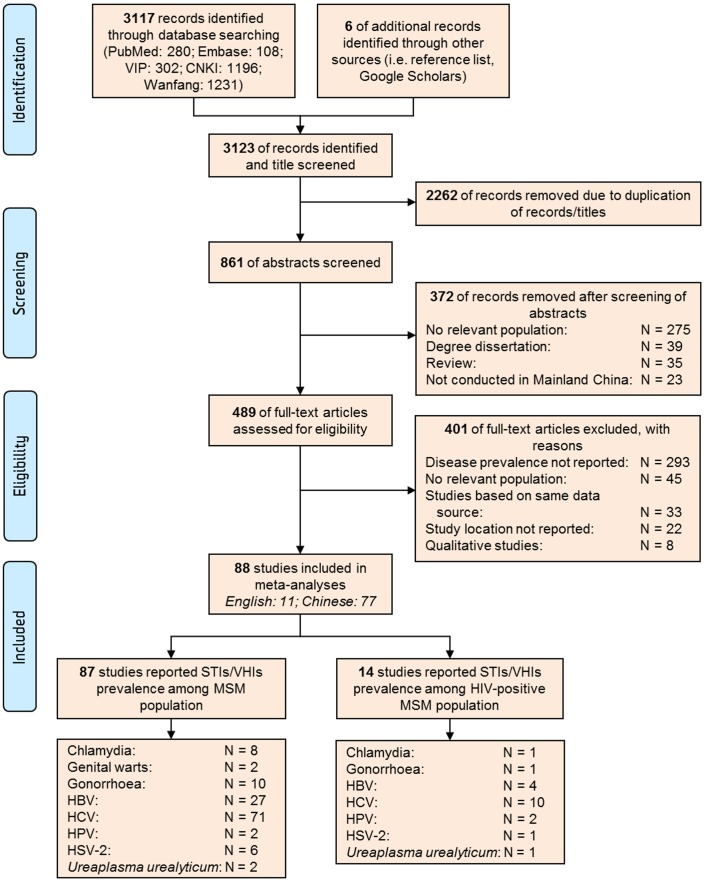
PRISMA flow chart for selection of studies. N represents the number of studies identified.

We identified 3,123 records based on our search strategy, of which 861 were unique records. After the initial screening of abstracts, we reviewed 489 articles in full and 401 articles were excluded based on our selection criteria. A total of 88 studies were included (Figure 1). The sample size of MSM in the eligible studies ranged from 59 to 1,462 (median: 273; interquartile range: 156–456) and the overall sample consisted of 35,203 MSM. The weighted mean age of MSM was 27.4 years (range: 14–84 years). The majority of studies were cross-sectional observational studies (*n* = 85); but two were before-and-after studies and one was cohort study. Seventy-one studies reported on HCV, twenty-seven on HBV, ten on gonorrhoea, eight on chlamydia, seven on HSV-2, two on HPV and genital wart, respectively. Most of studies (*n* = 23) recruited study participants at MSM hotspot venues; however, the majority of remaining studies (*n* = 43) utilised a combination of recruitment methods (Table S1). Fourteen studies reported the prevalence of STIs among HIV-positive MSM (Table S2).

### Hepatitis Infections

**Figure 2:**
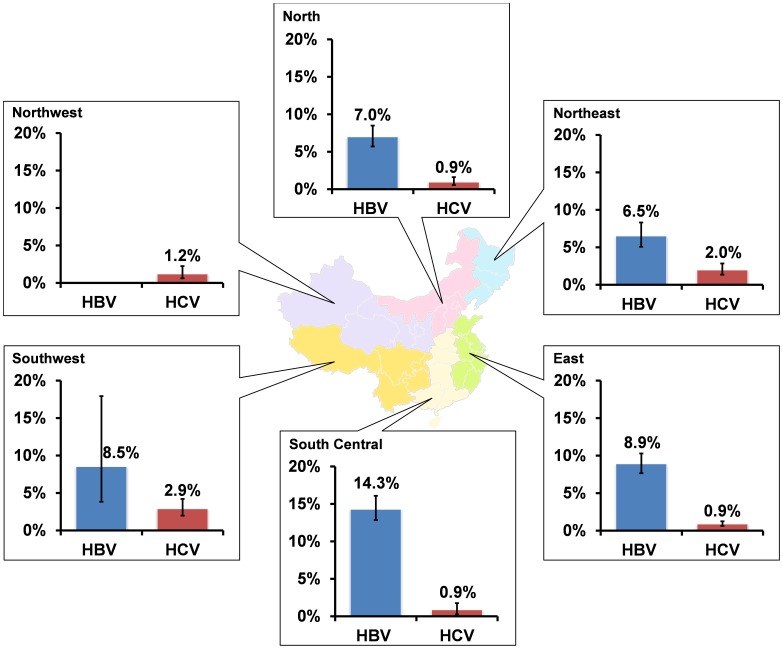
Prevalence of HBV and HCV infection among MSM in six Chinese regions.

The estimated HBV and HCV prevalence among MSM were 8.9% (95% CI: 7.8–10.2%) (Figure S1) and 1.2% (1.0–1.6%) (Figure S2) over the period 2003–2011 and heterogeneities across the studies were substantial (HBV: χ^2^ = 166.0, *p*<0.001; *I* ^2^ = 79.5; HCV: χ^2^ = 335.4, *p*<0.001; *I* ^2^ = 76.2) (Table 1, Table S3). Study location stratification substantially reduced heterogeneities (Table S3). HBV prevalence varied extensively across geographical regions (χ^2^ = 56.6, *p*<0.001), ranged from 6.5% (5.1–8.3%) in the Northeast to 14.3% (12.6–16.2%) in the South Central (Figure 2). No HBV prevalence was reported in the Northwest. In comparison, South Central had the lowest HCV prevalence (0.9% [0.5–1.5%]); while prevalence in the Southwest was the highest (2.9% [2.0–4.2%]) (Figure 2). Among HIV-positive MSM, 18.3% (9.8–31.5%) and 8.4% (3.9–17.3%) were co-infected with HBV and HCV, respectively. The temporal trends of HBV (*p* = 0.212) and HCV (*p* = 0.321) prevalence among MSM were not significant (Figure S3).

**Table 1:** Meta-analyses of STIs and hepatitis infections prevalence among broader MSM comparing among HIV-positive MSM and male sex workers in China.

Diseases Burden	MSM population (Ref)			HIV-positive MSM				Male sex workers			
	Number ofstudies (numberof estimates)φ	Totalsamplesize	EstimatedPrevalence(95% CI)	Number ofstudies(number ofestimates)	Total samplesize of HIV-positive	EstimatedPrevalence(95% CI)	Odds ratio(95% CI)∧	Number ofstudies(number ofestimates)	Totalsamplesize	EstimatedPrevalence(95% CI)	Odds ratio(95% CI)∧
**Hepatitis infections**											
HBV	27 (35) [56,61,65,86,121–143]	11,305	8.9%(7.8–10.2%)	4 (4) [56,121,139,144]	151	18.3%(9.8–31.5%)	2.3(1.5–3.5)***	1(1) [145]	120	4.2%(1.7–9.6%)	0.4(0.2–1.1)
HCV	71 (81) [55,56,61,65,79,86,121,122,124–126,128–142,146–189]	28,684	1.2%(1.0–1.6%)	10 (10) [55,121,128,139,144,152,162,178,179,183]	363	8.4%(3.9–17.3%)	7.6(5.2–11.2)***	2(2) [145], [190]	625	2.7%(0.6–12.0%)	2.2(1.4–3.7)**
**Sexually transmitted infections**											
Chlamydia	8 (12) [55,133,140,141,191–194]	3,921	6.3%(3.5–11.0%)	1 (1) [55]	16	31.3%(13.6–56.7%)	6.7(2.3–19.5) ***	1(1) [195]	82	14.6%(8.5–24.0%)	2.5(1.4–4.7) **
*Sera*	1 (1) [55]	753	5.6%(4.15–7.5%)	–	–	–	–	–	–	–	–
*Urethral*	5 (8) [133,140,141,191,192]	2,411	4.3%(2.5–7.5%)	–	–	–	–	–	–	–	–
*Rectal*	3 (3) [192–194]	757	16.5%(6.9–34.4%)	–	–	–	–	–	–	–	–
Genital warts	2 (3) [56], [79]	581	1.5%(0.7–2.9%)	–	–	–	–	–	–	–	–
Gonorrhoea	10 (13) [56,79,133,140,191–194,196,197]	3,668	1.9%(1.3–2.7%)	1 (1) [56]	24	4.2%(0.6–24.4%)	2.3(0.3–17.2)	1(1) [195]	82	9.8%(5.0–18.3%)	5.7(2.7–12.3) ***
*Sera*	1 (1) [197]	157	2.6%(1.0–6.6%)	–	–	–	–	–	–	–	–
*Urethral*	4 (4) [133,140,191,194]	891	1.4%(0.8–2.7%)	–	–	–	–	–	–	–	–
*Rectal*	3 (3)[140], [193], [194]	687	1.0%(0.1–7.2%)	–	–	–	–	–	–	–	–
HIV#	N/A (60) [42]	20,729	4.7%(3.9–5.6%)	–	–	–	–	16 (16) [42]	2,278	6.0%(4.2–8.5%)	1.3(1.1–1.6) **
HPV											
*Any type*	2 (2)[198], [199]	861	66.3%(57.4–74.1%)	2 (2) [198], [199]	79	96.2%(88.9–98.8%)	12.9(4.0–41.0) ***	–	–	–	–
*Single type*	2 (2)[198], [199]	861	32.8%(29.7–36.0%)	2 (2) [198], [199]	79	40.5%(30.3–51.7%)	1.4(0.9–2.2)	–	–	–	–
*Multiple types*	2 (2)[198], [199]	861	33.4% (25.3–42.6%)	2 (2) [198], [199]	79	56.9%(45.8–67.4%)	2.6(1.7–4.2) ***	–	–	–	–
*HPV16*	2 (2)[198], [199]	861	8.0%(2.8–20.9%)	1 (1) [198]	50	34.0%(22.3–48.1%)	5.9(3.1–11.1) ***	–	–	–	–
*HPV18*	1 (1) [198]	578	5.9%(4.2–8.1%)	1 (1) [198]	50	14.0%(6.8–26.6%)	2.6(1.1–6.2) **	–	–	–	–
*HPV45*	2 (2)[198], [199]	861	4.4%(2.7–7.2%)	1 (1) [198]	50	14.0%(6.8–26.6%)	3.5(1.5–8.3) **	–	–	–	–
HSV-2	6 (6) [24,84,133,189,200,201]	3,451	10.6%(6.2–17.6%)	1 (1) [24]	47	33.8%(26.4–42.1%)	4.3(2.3–7.9) ***	3(3) [202]–[204]	598	10.0%(7.6–12.9%)	0.9(0.7–1.2)
Syphilis#	N/A(40) [42]	15,317	13.5%(11.8–15.3%)	N/A (8) [42]	317	36.2%(24.3–50.0%)	3.6(2.9–4.6) ***	15 (15) [42]	2,237	12.4%(9.9–15.3%)	0.9(0.8–1.0)
*Ureaplasma* *urealyticum*	2 (2)[55], [205]	938	4.3%(3.2–5.8%)	1 (1) [198]	16	18.8%(6.2–44.8%)	5.2(1.4–18.9) *	–	–	–	–

φ Some studies reported more than one prevalence estimate (e.g. in multiple years or locations). The number of estimates represents the total number of data points analysed in meta-analysis.

# The estimated prevalence for HIV, syphilis and HIV-syphilis co-infection were extracted from a published meta-analysis study by Chow EPF *et al* in 2011.

∧ The broader MSM population was used as the reference group for odds ratio.

*p<0.05, **p<0.01, ***p<0.001.

The risks of HBV and HCV infections among MSM were significantly higher than general Chinese adults (HBV: OR = 1.4 [1.3–1.6]; HCV: OR = 10.1 [4.5–22.8]) (Table 2). Further, the risk of HCV infection was 2.2 (1.4–3.7) times higher among MSW compared with broader MSM, but the risks of HBV infection were similar (OR = 0.4 [0.2–1.1]). Among HIV-positive MSM, the risks of HBV and HCV infection were 2.3 (1.5–3.5) and 7.6 (5.2–11.2) times higher than the broader MSM population (Table 1).

**Table 2:** Comparison of hepatitis infections and STIs prevalence among MSM and adults in general population in China.

Diseases burden	Adults in general population (Ref)			MSM
	Total sample size	Estimated prevalence (%)	Sources	Odds ratio (95% CI)∧
Chlamydia	6,334	3.0% (2.2–4.1%)	[206]–[208]	2.2 (1.8–2.6)***
Gonorrhoea	5,366	0.2% (0.1–0.4%)	[206], [208]	13.0 (6.3–27.1)***
HIV	52,601	0.05% (0.04–0.08%)	[8]	98.7 (64.3–136.4)***
HBV	20,403	6.4% (4.5–9.1%)	[209]–[211]	1.4 (1.3–1.6)***
HCV	4,950	0.1% (0.1–0.3%)	[212]	10.1 (4.5–22.8)***
Syphilis	17,226	0.5% (0.3–0.7%)	[213], [214]	29.9 (24.1–36.9)***

∧ The general population was used as the reference group for odds ratio.

*p<0.05, ** *p*<0.01, *** *p*<0.001.

### Sexually Transmitted Infections

Previous meta-analysis study reported the prevalence of HIV and syphilis among MSM to be 4.7% (3.9–5.6%) and 13.5% (11.8–15.3%), respectively [11]. Approximately 36.2% (24.3–50.0%) of HIV-positive MSM were co-infected with syphilis (Table 1).

The pooled prevalence of chlamydia was 6.3% (3.5–11.0%) (Figure S4) and heterogeneity was significant (χ^2^ = 223.6, *p*<0.001; *I* ^2^ = 95.1). Subgroup analyses indicated that the prevalence substantially varied by anatomical site, ranged from 4.3% (2.5–7.5%) for urethral chlamydia to 16.5% (6.9–34.4%) for rectal chlamydia. A single study estimated that 31.3% of HIV-positive MSM were also co-infected with chlamydia [55].

The overall sero-prevalence of gonococcal infection was 1.9% (1.3–2.7%), and heterogeneity was significant (χ^2^ = 51.0, *p*<0.001; *I* ^2^ = 74.5) (Figure S5). Stratified analysis according to the anatomical site of the infection substantially reduced heterogeneity (urethral: *I* ^2^ = 67.6; rectal: *I* ^2^ = 5.8) (Table S3). A lower overall prevalence was observed in rectal gonorrhoea (1.0% [0.1–7.2%]) than that of urethral gonorrhoea (1.4% [0.8–2.7%]). One study reported 4.2% of HIV-positive MSM were co-infected with gonorrhoea [56].

The pooled prevalence of HPV infection was 66.3% (57.4–74.1%) and the heterogeneity was substantial (χ^2^ = 6.1, *p* = 0.014; *I* ^2^ = 83.6) (Figure S6). Subgroup analyses showed that about 32.8% (29.7–36.0%) of Chinese MSM were single type infected and 33.4% (25.3–42.6%) were multiple types infected. Genotype HPV16 (8.0% [2.8–20.9%]) was found to be the most frequently identified sub-type, and was followed by HPV18 (5.9% [4.2–8.1%]) and HPV45 (4.4% [2.7–7.2%]). Co-infection between HIV and any genotypes of HPV genotypes was common (96.2% [88.9–98.8%]).

The pooled estimate of HSV-2 prevalence was 10.6% (6.2–17.6%) with high heterogeneity (χ^2^ = 126.8, *p*<0.001; *I* ^2^ = 96.1) across the included studies (Figure S7). Among HIV-positive MSM, estimated 33.8% were co-infected with HSV-2. The pooled prevalence of genital wart and *Ureaplasma urealyticum* were 1.5% (0.7–2.9%) and 4.3% (3.2–5.8%), respectively (Figure S8–S9).

In comparison with the general Chinese population, infection risk of STIs was significantly higher in MSM (chlamydia: OR = 2.2 [1.8–2.6]; gonorrhoea: 13.0 [6.3–27.1]; syphilis: 29.9 [24.1–36.9], Table 2). On contrast, MSW were subjected to even higher risk of STIs. The odds of chlamydial and gonococcal infections were 2.5 (1.4–4.7) and 5.7 (2.7–12.3) times higher in MSW compared with MSM, but the risks of syphilis and HSV-2 infections did not differ (Table 1). Similarly, HIV-positive MSM also demonstrated consistently greater risk of STIs compared with broader MSM (chlamydia: OR = 6.7 [2.3–19.5]; HPV: 12.9 [4.0–41.0]; HSV-2: 4.3 [2.3–7.9]; syphilis: 3.6 [2.9–4.6]; and *Ureaplasma urealyticum*: 5.2 [1.4–18.9]).

### Risk of Bias within and Across Studies

Among the 88 included studies, seven were at low risk for all six methodological quality items (Figure S10). The remaining 81 studies were at high or unclear risk of at least one of the bias items but none of them was at high risk for all the items. Overall, most studies had low risk of methods for selecting study participants, methods for measuring exposure and outcome variables, and the statistical methods used (Figure S11). Substantial publication bias was found among studies reported HCV prevalence estimates (*p* = 0.045), but not those reported chlamydia (*p* = 0.131), genital wart (*p* = 0.602), gonorrhoea (*p* = 0.154) and HBV (*p* = 0.081) (Table S3).

## Discussion

This study illustrates the complexity, heterogeneity and diversity of STIs and hepatitis infections among MSM in China. Our findings strongly indicate disproportionately high prevalence levels of STIs among Chinese MSM. The prevalence levels of STIs among HIV-positive MSM and MSW appeared to be higher than the broader MSM population.

Our findings illustrated that the transmission of HBV and HCV among Chinese MSM has distinct geographically patterns. HBV prevalence in China is 8.9%, which is higher than developed countries such as Australia (3%) [57], and the USA (4%) [58]. South Central region has the highest HBV prevalence across the country (14.3%). This is similar to patterns in the general population which major cities in South Central have consistently higher HBV prevalence (10.4–12.5% [59], [60]) than other Chinese regions (e.g. 3.5% in Beijing [61]). HBV transmission in China is primarily attributed to perinatal transmission [62]–[64]. Individuals engaging in high-risk behaviours, in particular, MSM with a history of STIs, injecting drug use and commercial sex activities, have elevated risk of HBV infection [65]–[70]. Although vaccination program for hepatitis B has been expanded through the National Expanded Program on Immunisation (EPI) in 1992 [71] and free hepatitis B vaccines have been provided to all newborns since 2005 [72], the majority (∼61%) of Chinese MSM remains unvaccinated, especially for those above age of 30 [65]. Low HBV immunisation coverage rate among MSM is not only found in China (∼39%), but is also observed in developed countries such as the USA (25%) [68], and Australia (53%) [73]. Successful HBV vaccination program integration as part of the sexual health programs have been observed globally [74]–[76], similar strategy especially targeting MSM are recommended in China [65], [72]. In comparison, HCV prevalence pattern in MSM is similar to that of drug use in China [77], [78]. Southwest China has a distinctly greater HCV disease burden (2.89%) in comparison with other regions. Notably 1–6% of Chinese MSM are also injecting drug users [79]–[85]. However, the risk of HCV infection may be further elevated by their high-risk homosexual behaviours [86]–[89]. The recently reported HCV outbreaks among HIV-infected but non-injecting MSM in developed countries strongly indicate that HIV-infection and high-risk sexual behaviours may also contribute to HCV transmission in MSM [90], [91]. Behavioural interventions among MSM, especially HIV-positive MSM, remain a priority for prevention of STIs and hepatitis for MSM.

The consistently much higher risks of STIs and hepatitis infections among MSM are not unexpected but alarming. Many STIs, such as syphilis, HPV, chlamydia and gonorrhoea, are known to cause genital ulceration and inflammation which may significantly increase the infection risk of HIV, HBV, HCV and other STIs [92]–[99]. Co-infections of STIs and hepatitis infections also substantially complicated the provision of care and treatment for infected individuals, especially among those living with HIV [100]–[103]. HPV is the most prevalent STIs among Chinese MSM (66%), this result is consistent with the findings in countries such as Netherlands (72%) [104], Slovenian (78%) [105], and Australia (79%) [106]. In particular, our findings indicate that the odds of HPV infection among HIV-positive MSM was the highest (OR = 12.9) among the included STIs. HPV is known to be the primary etiological agent for anal cancer [107], the progression rate of high-grade anal intraepithelial neoplasia to anal carcinoma among HIV-positive MSM (1/600 per year) is much faster than that of HIV-negative MSM (1/4000 per year), suggesting HIV-positive MSM have a much higher risk of anal caner [22]. HPV also substantially facilitate the transmission of other STIs such as chlamydia, gonorrhoea [108] and HSV-2 [109], leading to complications in prevention and treatment of these STIs.

This study has a number of limitations. First, the majority of the studies included in this review employed non-probabilistic sampling approaches to recruit MSM, and studies were mostly conducted at gay-oriented venues in large urban cities. This limits the generalisability of the study as MSM who frequently attended gay venues, particularly gay saunas and bars, were known to have greater high-risk behaviours and risks of HIV and STIs [110], [111]. Second, as ∼90% of the Chinese MSM have engaged in oral sex with other men [12], [112]–[116] and more than half of these episodes (∼55%) were not protected by condoms [12], oral transmission of STIs is likely to be common and asymptomatic in MSM. However, pharyngeal infections of STIs were examined in none of our collected studies. Third, the number of studies on HIV-positive MSM are limited and the sample sizes are generally small (mean = 45.7, range = 12–149), limiting the statistical power of meta-analysis. Fourth, this study did not investigate the geographical and temporal trends of STIs among MSM due to insufficient data. Fifth, clinical diagnostic algorithms of STIs and hepatitis infections vary in different studies and study time points, which may lead to uncertainties in its sensitivity and specificity. The pooled estimates of STIs and hepatitis infections must be interpreted with cautions. Sixth, demographic characteristics (such as age, marital status, level of education) and psychosocial factors are also associated with STIs acquisition; however, we were not able to investigate these factors due to insufficient data reported in the eligible articles.

A number of priorities should be noted in response to the high disease burdens of STIs among Chinese MSM. First, an effective integration of HIV and STIs healthcare and surveillance systems is required. Although diagnosis and treatment of HIV and STIs share many common grounds, China has two parallel systems operating to provide counselling, testing and treatment for people living with HIV and STIs respectively [34], [117], [118]. As HIV-positive MSM are commonly infected with other STIs, timely referral and follow-up of HIV-positive MSM to STIs screening and treatment is necessary. Second, HPV is the most prevalence infection among all STIs in Chinese MSM. Although clinical use of quadrivalent HPV vaccine can effectively reduce the HPV infection among MSM [119], currently no country has a universal HPV vaccination program specifically targeting MSM [120]. Finally, expansion of HBV vaccination and screening and treatment for HCV-positive MSM are also priorities for curbing the viral hepatitis epidemics and reducing complications in co-infection treatments for this HIV high-risk group in China.

## Supporting Information

Figure S1 **Prevalence of hepatitis B virus infection among Chinese MSM.** Forest plots showing unadjusted prevalence estimates (squares) with 95% confidence intervals (lines). Pooled prevalence estimate is presented as rhombus in this plot.
(PDF)

Figure S2 **Prevalence of hepatitis C virus infection among Chinese MSM.** Forest plots showing unadjusted prevalence estimates (squares) with 95% confidence intervals (lines). Pooled prevalence estimate is presented as rhombus in this plot.
(PDF)

Figure S3 **Temporal trends of HBV and HCV prevalence among MSM in China.**
(PDF)

Figure S4 **Prevalence of chlamydia among Chinese MSM.** Forest plots showing unadjusted prevalence estimates (squares) with 95% confidence intervals (lines). Pooled prevalence estimate is presented as rhombus in this plot.
(PDF)

Figure S5 **Prevalence of gonorrhoea among Chinese MSM.** Forest plots showing unadjusted prevalence estimates (squares) with 95% confidence intervals (lines). Pooled prevalence estimate is presented as rhombus in this plot.
(PDF)

Figure S6 **Prevalence of HPV infection among Chinese MSM.** Forest plots showing unadjusted prevalence estimates (squares) with 95% confidence intervals (lines). Pooled prevalence estimate is presented as rhombus in this plot.
(PDF)

Figure S7 **Prevalence of HSV-2 infection among Chinese MSM.** Forest plots showing unadjusted prevalence estimates (squares) with 95% confidence intervals (lines). Pooled prevalence estimate is presented as rhombus in this plot.
(PDF)

Figure S8 **Prevalence of genital wart (** ***Condyloma acuminatum*** **) infection among Chinese MSM.** Forest plots showing unadjusted prevalence estimates (squares) with 95% confidence intervals (lines). Pooled prevalence estimate is presented as rhombus in this plot.
(PDF)

Figure S9 **Prevalence of** ***Ureaplasma urealyticum*** **infection among Chinese MSM.** Forest plots showing unadjusted prevalence estimates (squares) with 95% confidence intervals (lines). Pooled prevalence estimate is presented as rhombus in this plot.
(PDF)

Figure S10 **Risk of bias summary: review authors’ judgements about each risk of bias item for each included study.**
(PDF)

Figure S11 **Risk of bias graph: review authors’ judgements about each risk of bias item presented as percentages across all included studies.**
(PDF)

Table S1 **Systematic review of 87studies reporting the prevalence of sexually transmitted infections and/or viral hepatitis infections among men who have sex with men in China.**
(DOC)

Table S2 **Systematic review of 14 studies reporting the co-infection prevalence of sexually transmitted infections and/or viral hepatitis infections among HIV-positive men who have sex with men in China.**
(DOC)

Table S3 **Publication bias and heterogeneity in subgroup meta-analyses.**
(DOC)

Checklist S1 **PRISMA checklist.**
(DOC)
