# Supplementary figures and images for: Disparities and Risks of Sexually Transmissible Infections among Men Who Have Sex with Men in China: A Meta-Analysis and Data Synthesis
Source: PLoS One. 2014 Feb 24;9(2):e89959. doi: 10.1371/journal.pone.0089959 (PMC3933676; doi:10.1371/journal.pone.0089959)

**Figure S1. Prevalence of hepatitis B virus infection among Chinese MSM.**

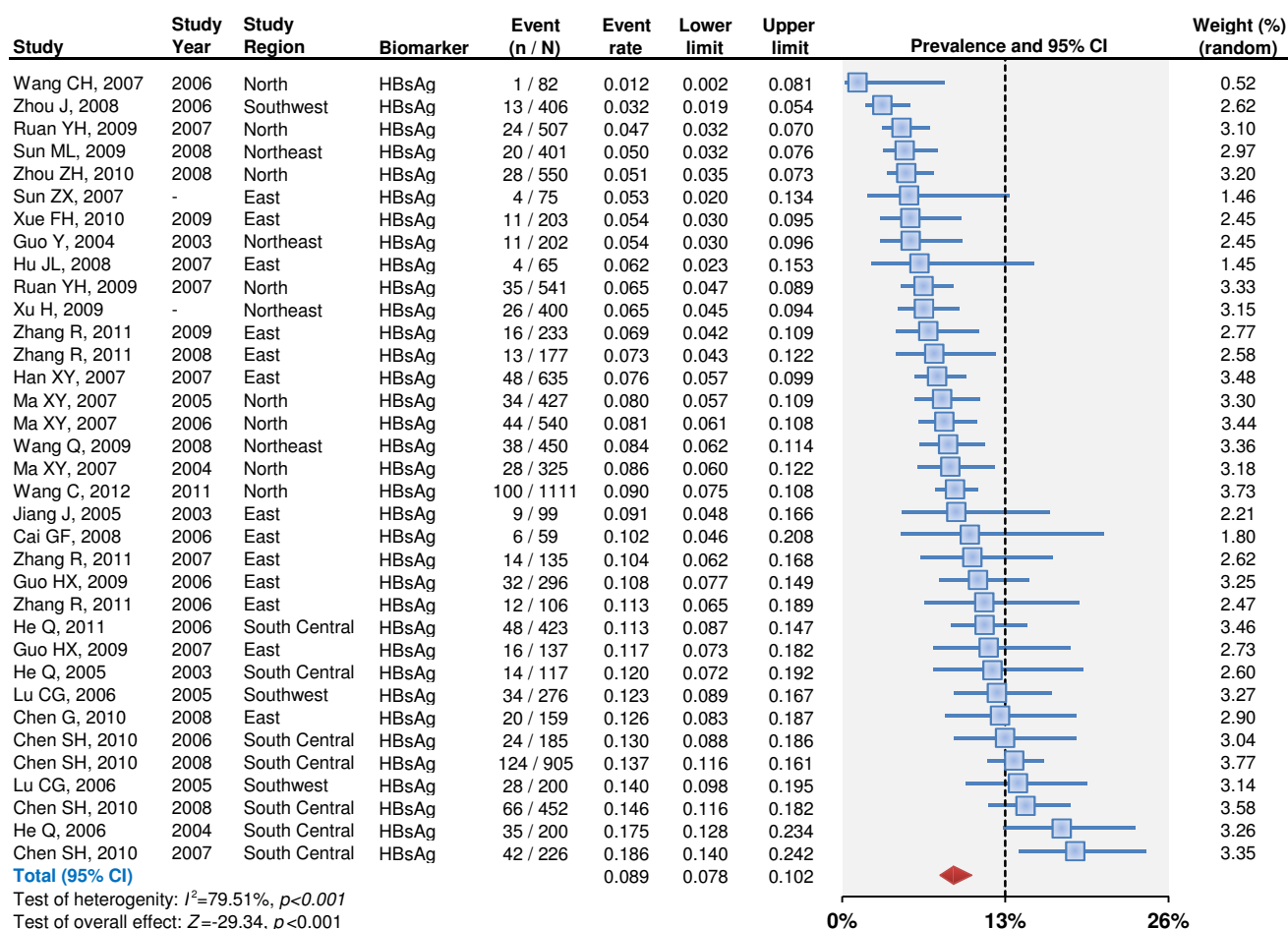

Supplement: Figure S1 — Prevalence of hepatitis B virus infection among Chinese MSM. Forest plots showing unadjusted prevalence estimates (squares) with 95% confidence intervals (lines). Pooled prevalence estimate is presented as rhombus in this plot. (PDF) [file pone.0089959.s001.pdf]

**Figure S2. Prevalence of hepatitis C virus infection among Chinese MSM.**

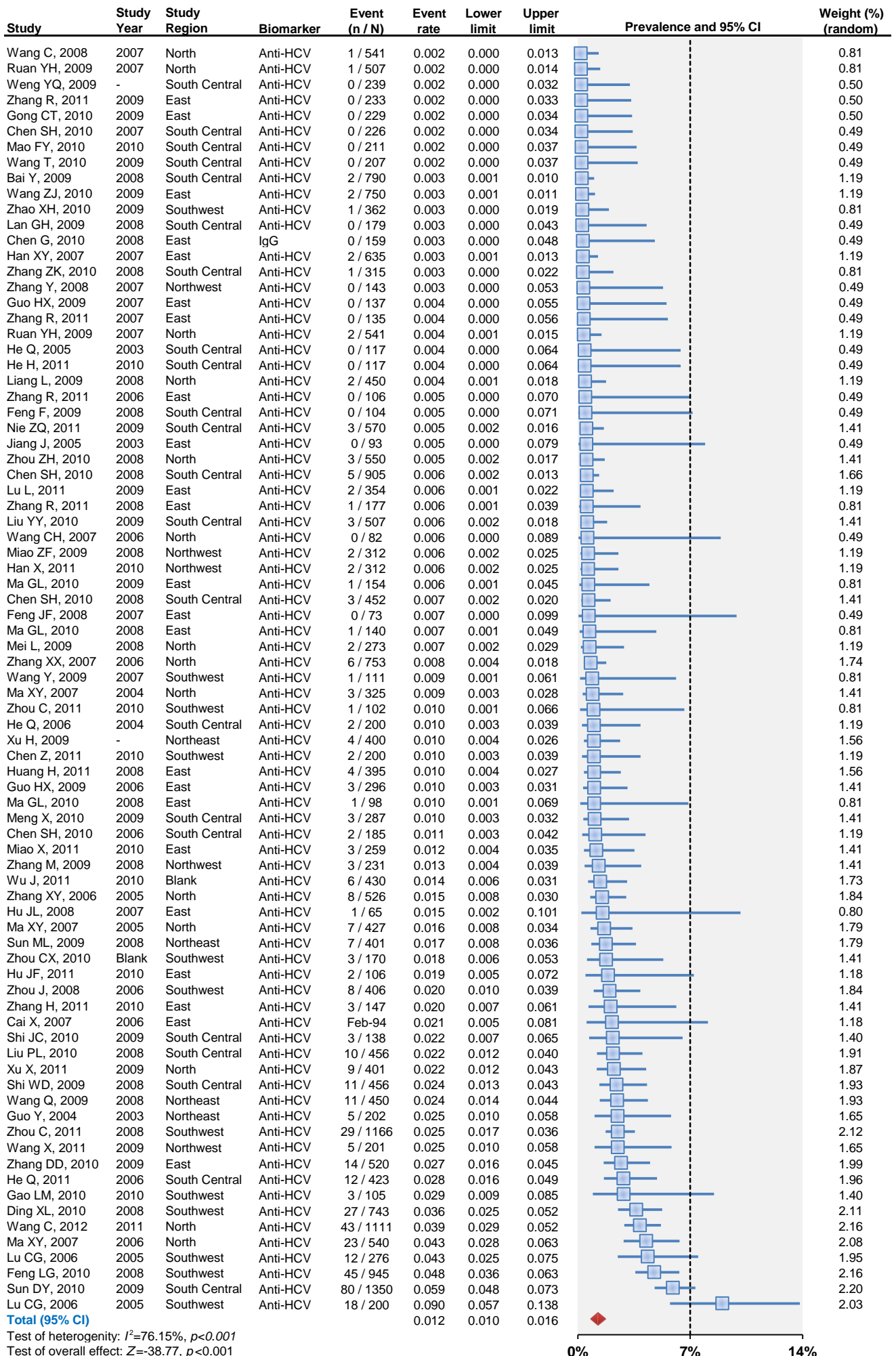

Supplement: Figure S2 — Prevalence of hepatitis C virus infection among Chinese MSM. Forest plots showing unadjusted prevalence estimates (squares) with 95% confidence intervals (lines). Pooled prevalence estimate is presented as rhombus in this plot. (PDF) [file pone.0089959.s002.pdf]

Figure S3. Temporal trends of HBV and HCV prevalence among MSM in China

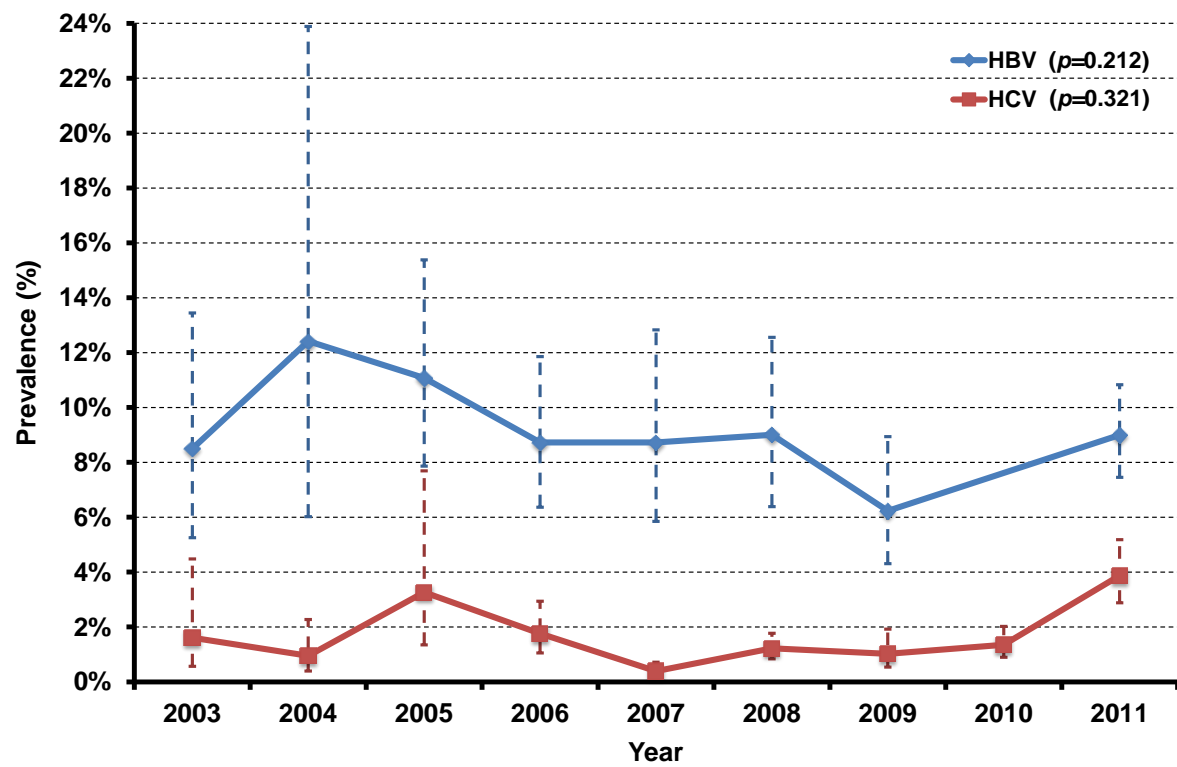

Supplement: Figure S3 — Temporal trends of HBV and HCV prevalence among MSM in China. (PDF) [file pone.0089959.s003.pdf]

**Figure S4. Prevalence of chlamydia among Chinese MSM.**

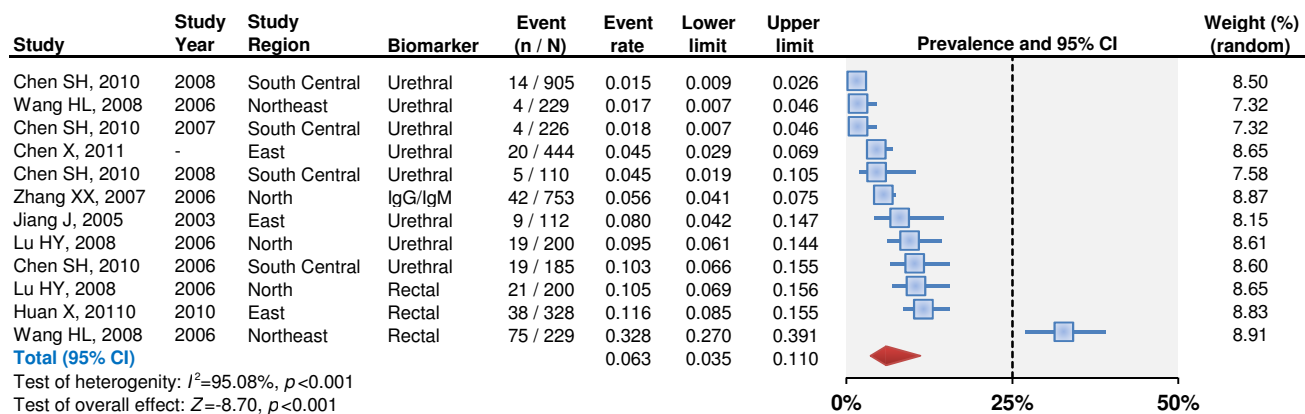

Supplement: Figure S4 — Prevalence of chlamydia among Chinese MSM. Forest plots showing unadjusted prevalence estimates (squares) with 95% confidence intervals (lines). Pooled prevalence estimate is presented as rhombus in this plot. (PDF) [file pone.0089959.s004.pdf]

**Figure S5. Prevalence of gonorrhoea among Chinese MSM.**

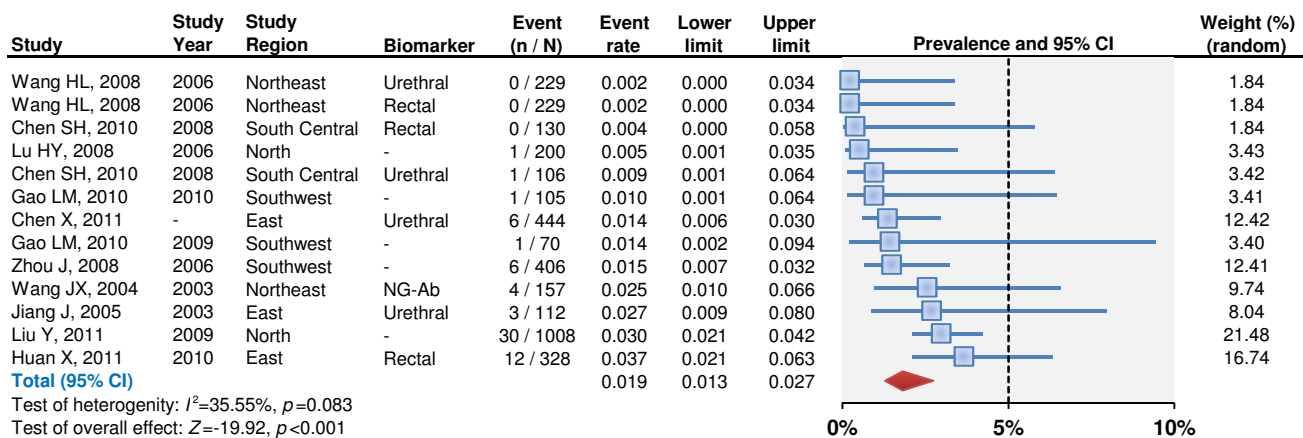

Supplement: Figure S5 — Prevalence of gonorrhoea among Chinese MSM. Forest plots showing unadjusted prevalence estimates (squares) with 95% confidence intervals (lines). Pooled prevalence estimate is presented as rhombus in this plot. (PDF) [file pone.0089959.s005.pdf]

Figure S6. Prevalence of HPV infection among Chinese MSM.

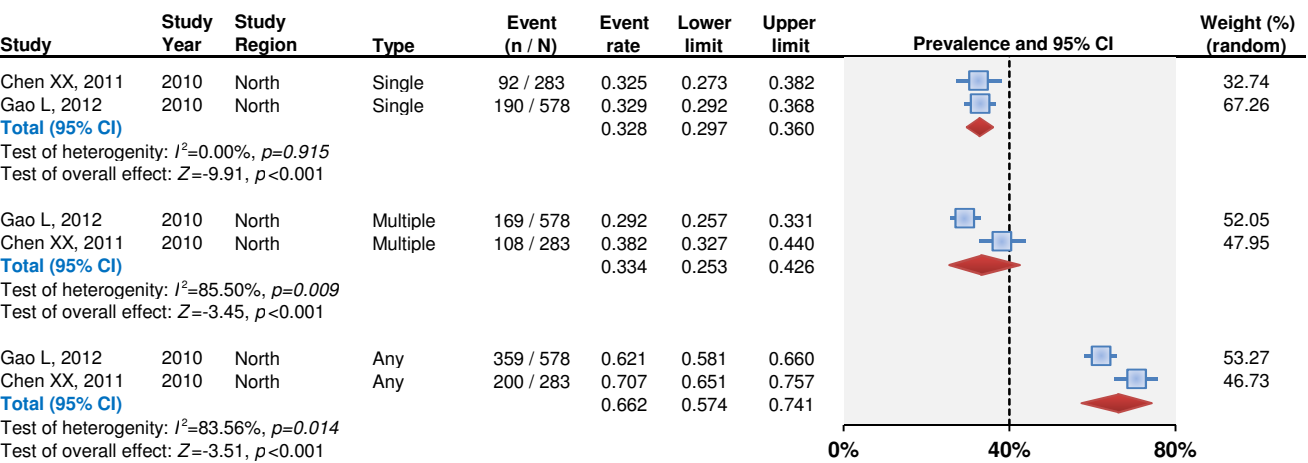

Supplement: Figure S6 — Prevalence of HPV infection among Chinese MSM. Forest plots showing unadjusted prevalence estimates (squares) with 95% confidence intervals (lines). Pooled prevalence estimate is presented as rhombus in this plot. (PDF) [file pone.0089959.s006.pdf]

**Figure S7. Prevalence of HSV-2 infection among Chinese MSM.**

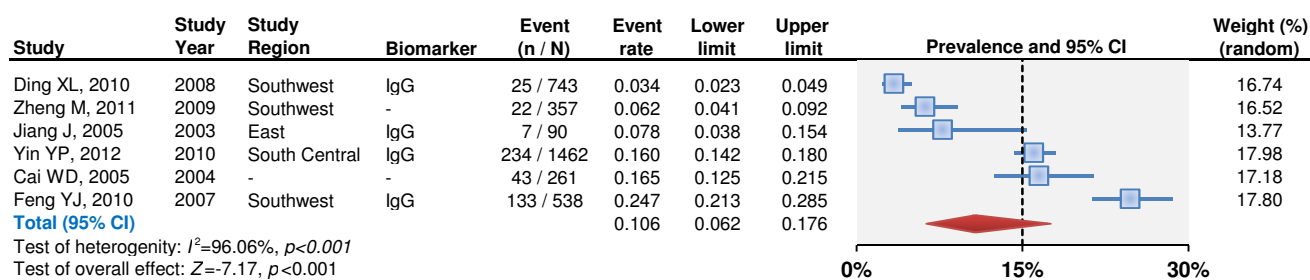

Supplement: Figure S7 — Prevalence of HSV-2 infection among Chinese MSM. Forest plots showing unadjusted prevalence estimates (squares) with 95% confidence intervals (lines). Pooled prevalence estimate is presented as rhombus in this plot. (PDF) [file pone.0089959.s007.pdf]

**Figure S8. Prevalence of genital warts (*Condyloma acuminatum*) infection among Chinese MSM.**

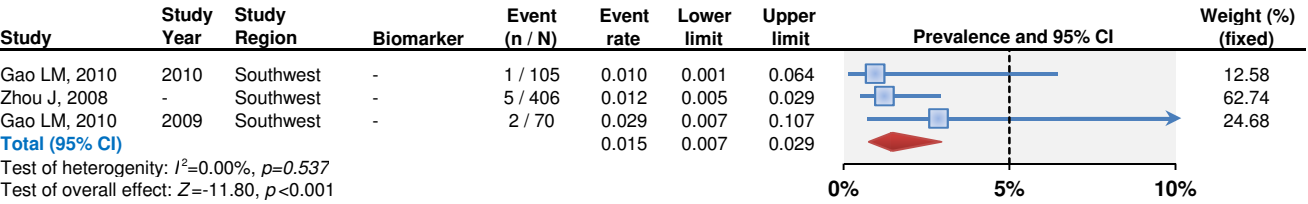

Supplement: Figure S8 — Prevalence of genital wart ( Condyloma acuminatum ) infection among Chinese MSM. Forest plots showing unadjusted prevalence estimates (squares) with 95% confidence intervals (lines). Pooled prevalence estimate is presented as rhombus in this plot. (PDF) [file pone.0089959.s008.pdf]

Figure S9. Prevalence of *Ureaplasma urealyticum* infection among Chinese MSM.

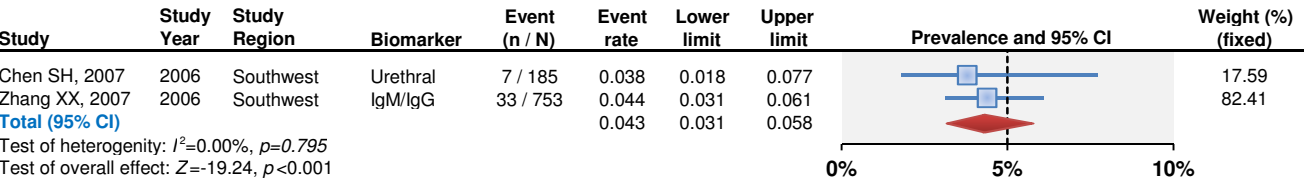

Supplement: Figure S9 — Prevalence of Ureaplasma urealyticum infection among Chinese MSM. Forest plots showing unadjusted prevalence estimates (squares) with 95% confidence intervals (lines). Pooled prevalence estimate is presented as rhombus in this plot. (PDF) [file pone.0089959.s009.pdf]
