# Supplementary material for: Disparities and Risks of Sexually Transmissible Infections among Men Who Have Sex with Men in China: A Meta-Analysis and Data Synthesis
Source: PLoS One. 2014 Feb 24;9(2):e89959. doi: 10.1371/journal.pone.0089959 (PMC3933676; doi:10.1371/journal.pone.0089959)
Supplement: Figure S10 — Risk of bias summary: review authors’ judgements about each risk of bias item for each included study. (PDF) [file pone.0089959.s010.pdf]

Figure S10. Risk of bias summary: review authors' judgements about each risk of bias item for each included study.

Study	Methods for selecting study participants	Methods for measuring exposure and outcome variables	Design-specific sources of bias (excluding confounding)	Method of control confounding	Statistical methods (excluding control of confounding)	Other biases (i.e. conflict of interest, funding sources)
Bai Y, 2009	−	+	+	−	+	?
Cai GF, 2008	+	+	?	?	+	?
Cai WD, 2005	+	+	?	?	+	+
Cai X, 2007	−	?	?	?	+	?
Chen G, 2010	+	+	+	?	+	+
Chen SH, 2007	−	+	?	?	+	?
Chen SH, 2010	−	+	?	?	+	?
Chen SH, 2010	−	+	?	?	+	+
Chen XM, 2011	−	−	+	−	+	+
Chen XX, 2011	−	+	+	+	+	?
Chen Z, 2011	?	+	?	?	+	?
Ding XB, 2010	?	−	?	?	−	?
Feng F, 2009	+	+	+	+	+	+
Feng JF, 2008	?	+	?	?	+	?
Feng LG, 2010	?	+	?	?	+	+
Feng Y, 2010	+	+	+	+	+	+
Gao L, 2012	+	+	+	+	+	+
Gao LM, 2010	−	−	−	?	+	?
Gong CT, 2010	+	+	?	?	+	?
Gu Y, 2004	−	−	?	?	+	+
Guo H, 2009	−	+	?	?	+	+
Han X, 2011	+	+	?	?	+	+
Han XY, 2007	−	+	?	?	+	?
He H, 2011	−	?	?	?	+	+
He Q, 2005	+	+	?	?	+	?
He Q, 2006	+	+	?	?	+	?
He Q, 2011	+	+	+	+	+	+
Hu JF, 2011	+	?	?	?	+	?
Hu JL, 2008	+	+	?	?	+	?
Huan XP, 20110	+	+	+	?	+	+
Huang HY, 2011	+	+	+	−	+	?
Jiang J, 2005	+	+	+	+	+	+
Lan GH, 2009	−	−	?	?	−	?
Liang L, 2009	+	?	?	?	+	?
Liu PL, 2010	?	+	?	?	+	?
Liu Y, 2011	+	−	+	+	−	+
Liu YY, 2010	−	+	?	?	+	+
Lu CG, 2006	?	−	?	?	−	?
Lu CG, 2006	+	?	?	?	+	?
Lu HY, 2008	?	+	?	?	+	+
Lu L, 2011	?	+	?	?	+	?
Ma GL, 2010	+	−	?	?	−	?
Ma X, 2007	+	+	+	+	+	+
Mao FY, 2010	−	+	?	?	+	?

Study	Methods for selecting study participants	Methods for measuring exposure and outcome variables	Design-specific sources of bias (excluding confounding)	Method of control confounding	Statistical methods (excluding control of confounding)	Other biases (i.e. conflict of interest, funding sources)
Mei L, 2009	?	+	?	?	+	?
Meng X, 2010	?	+	?	?	−	?
Miao XL, 2011	−	+	?	?	+	?
Miao ZF, 2009	−	−	?	?	−	?
Nie ZQ, 2011	?	−	?	?	?	?
Ruan Y, 2009	+	+	+	?	+	?
Ruan Y, 2009	+	+	+	+	+	?
Shi JC, 2010	−	+	?	?	−	?
Shi WD, 2009	?	+	?	?	+	?
Sun DY, 2010	+	+	?	?	+	?
Sun ML, 2009	+	+	?	?	+	?
Sun ZX, 2007	+	−	?	?	−	?
Wang C, 2008	−	−	+	?	−	?
Wang C, 2012	+	+	+	+	+	+
Wang CH, 2007	+	−	?	?	+	?
Wang HL, 2008	+	+	?	?	+	+
Wang JX, 2004	?	+	?	?	+	?
Wang Q, 2009	?	?	?	?	+	?
Wang T, 2010	−	+	+	+	+	+
Wang WH, 2011	+	+	?	+	+	+
Wang X, 2011	+	+	+	−	+	?
Wang Y, 2009	−	?	?	?	?	+
Wang ZJ, 2010	+	?	?	?	+	?
Weng YQ, 2009	?	−	?	?	+	?
Wu J, 2011	−	+	?	?	+	?
Xu H, 2009	?	+	?	?	+	?
Xu XY, 2011	−	?	?	?	?	?
Xue FH, 2010	+	+	?	?	+	+
Yin YP, 2012	+	+	+	+	+	?
Zhang DD, 2010	?	?	?	?	+	?
Zhang H, 2011	?	+	?	?	+	?
Zhang M, 2009	−	+	?	?	+	?
Zhang R, 2011	+	+	?	?	+	?
Zhang X, 2007	+	+	−	?	+	+
Zhang XY, 2006	−	+	+	−	+	?
Zhang Y, 2008	+	?	?	?	+	?
Zhang ZK, 2010	+	+	?	?	+	+
Zhao XH, 2010	−	−	+	−	+	?
Zheng M, 2011	−	+	?	?	+	+
Zhou C, 2011	?	?	+	+	+	+
Zhou CX, 2010	?	−	?	?	?	?
Zhou CX, 2011	?	−	?	?	+	?
Zhou J, 2008	+	?	?	?	+	+
Zhou ZH, 2010	+	−	?	?	+	+
