# Supplementary material for: Disparities and Risks of Sexually Transmissible Infections among Men Who Have Sex with Men in China: A Meta-Analysis and Data Synthesis
Source: PLoS One. 2014 Feb 24;9(2):e89959. doi: 10.1371/journal.pone.0089959 (PMC3933676; doi:10.1371/journal.pone.0089959)

Figure S11. Risk of bias graph: review authors' judgements about each risk of bias item presented as percentages across all included studies.

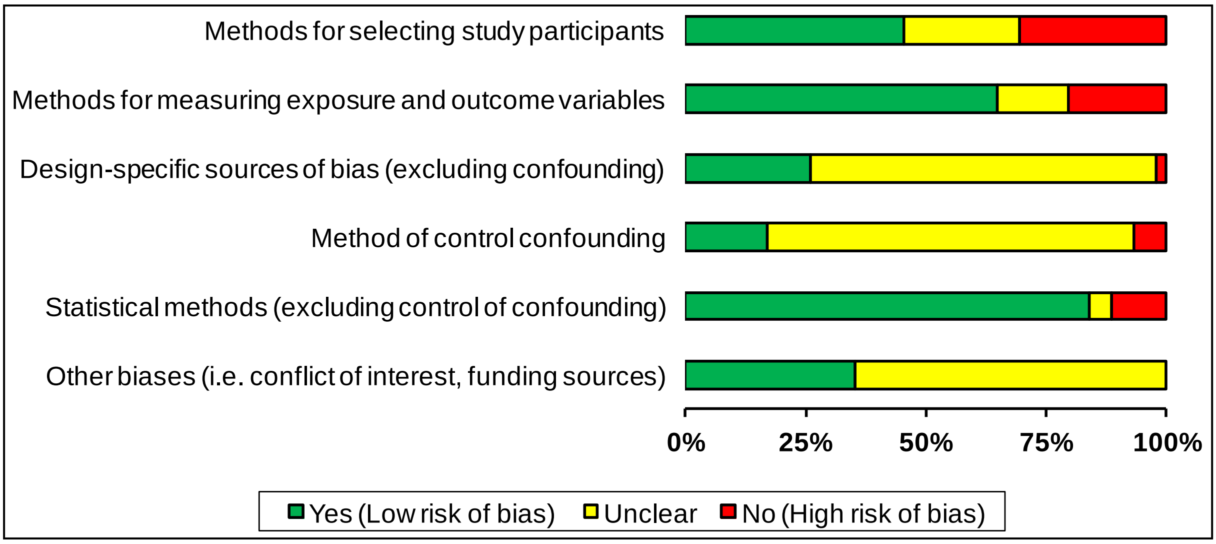

Supplement: Figure S11 — Risk of bias graph: review authors’ judgements about each risk of bias item presented as percentages across all included studies. (PDF) [file pone.0089959.s011.pdf]
