# Supplementary material for: Disparities and Risks of Sexually Transmissible Infections among Men Who Have Sex with Men in China: A Meta-Analysis and Data Synthesis
Source: PLoS One. 2014 Feb 24;9(2):e89959. doi: 10.1371/journal.pone.0089959 (PMC3933676; doi:10.1371/journal.pone.0089959)
Supplement: Table S1 — Systematic review of 87studies reporting the prevalence of sexually transmitted infections and/or viral hepatitis infections among men who have sex with men in China. (DOC) [file pone.0089959.s012.doc]

**Table S1**. Systematic review of 87 studies reporting the prevalence of sexually transmitted infections and/or viral hepatitis infections among men who have sex with men in China.

| **Study** | **Year** | **Province** | **Region**^#^ | **Age** | **Age Range** | **Study Design**^ϕ^ | **Recruitment method**^φ^ | **Sampling method**^δ^ | **Biomarker**^ | **Disease Prevalence** | | | |
| --- | --- | --- | --- | --- | --- | --- | --- | --- | --- | --- | --- | --- | --- |
|  |  |  |  |  |  |  |  |  |  | **Laboratory test of infection*** | **Sample size, N** | **n** | **Prevalence (%)** |
| ***a) Chlamydia*** | | | | | | | | | | | | | |
| Chen SH, 2010 | 2006 | Guangxi | SC | 26.22 | 14-68 | C-S | VCT | C, SB | Urethral | Emulsification | 185 | 19 | 10.27 |
| Chen SH, 2010 | 2007 | Guangxi | SC | 25.47 | 14-68 | C-S | VCT | C, SB | Urethral | Emulsification | 226 | 4 | 1.77 |
| Chen SH, 2010 | 2008 | Guangxi | SC | 27.35 | 14-68 | C-S | VCT | C, SB | Urethral | Emulsification | 905 | 14 | 1.55 |
| Chen SH, 2010 | 2008 | Guangxi | SC | 27.44 | 16-68 | C-S | VCT | SB | Urethral | Emulsification | 110 | 5 | 4.55 |
| Chen XM, 2011 | - | Jiangsu | E | 28.74 | 18-74 | C-S | - | RDS | Urethral | - | 444 | 20 | 4.50 |
| Huan XP, 2011 | 2010 | Jiangsu | E | 32.46 | - | C-S | MSM venues, internet advertisement | SB | Rectal | - | 328 | 38 | 11.59 |
| Jiang J, 2005 | 2003 | Jiangsu | E | - | 18-70 | C-S | MSM venues | C | Urethral swab | PCR | 112 | 9 | 8.04 |
| Lu HY, 2008 | 2006 | Beijing | N | 26.4 | 18-44 | C-S | CDC, peer referral | SB | Urethral | Cell culture | 200 | 19 | 9.50 |
| Lu HY, 2008 | 2006 | Beijing | N | 26.4 | 18-44 | C-S | CDC, peer referral | SB | Rectal | Cell culture | 200 | 21 | 10.50 |
| Wang HL, 2008 | 2006 | Liaoning | NE | - | 18-60 | C-S | - | - | Rectal | ELISA | 229 | 75 | 32.75 |
| Wang HL, 2008 | 2006 | Liaoning | NE | - | 18-60 | C-S | - | - | Urethral | ELISA | 229 | 4 | 1.75 |
| Zhang X, 2007 | 2006 | Beijing | N | 26.1 | 18-55 | C-S | VCT | PS | IgM/IgG | Assay Kit | 753 | 42 | 5.58 |
| ***b) Genital warts (Condyloma acuminatum)*** | | | | | | | | |  | | | | |
| Gao LM, 2010 | 2009 | Yunnan | SW | 36.3 | - | C-S | MSM venues, community outreach | - | - | - | 70 | 2 | 2.86 |
| Gao LM, 2010 | 2010 | Yunnan | SW | 33.3 | - | C-S | MSM venues, community outreach | - | - | - | 105 | 1 | 0.95 |
| Zhou J, 2008 | 2006 | Guizhou | SW | - | 15-49 | C-S | MSM venues, internet advertisement, telephone hotline | - | - | - | 406 | 5 | 1.23 |
| ***c) Gonorrhoea*** | | | | | | | | |  | | | | |
| Chen SH, 2010 | 2008 | Guangxi | SC | 27.44 | 16-68 | C-S | VCT | SB | Urethral | Cell culture | 106 | 1 | 0.94 |
| Chen SH, 2010 | 2008 | Guangxi | SC | 27.44 | 16-68 | C-S | VCT | SB | Rectal | Cell culture | 130 | 0 | 0.00 |
| Chen SH, 2010 | 2008 | Guangxi | SC | 27.44 | 16-68 | C-S | VCT | SB | Saliva | Cell culture | 144 | 16 | 11.11 |
| Chen XM, 2011 | - | Jiangsu | E | 28.74 | 18-74 | C-S | - | RDS | Urethral | - | 444 | 6 | 1.35 |
| Gao LM, 2010 | 2009 | Yunnan | SW | 36.3 | - | C-S | MSM venues, community outreach | - | - | - | 70 | 1 | 1.43 |
| Gao LM, 2010 | 2010 | Yunnan | SW | 33.3 | - | C-S | MSM venues, community outreach | - | - | - | 105 | 1 | 0.95 |
| Huan XP, 2011 | 2010 | Jiangsu | E | 32.46 | - | C-S | MSM venues, internet advertisement | SB | Rectal | - | 328 | 12 | 3.66 |
| Jiang J, 2005 | 2003 | Jiangsu | E | - | 18-70 | C-S | MSM venues | C | Urethral | PCR | 112 | 3 | 2.68 |
| Liu Y, 2011 | 2009 | Beijing | N | 27.6 | 16-59 | C-S | MSM venues, internet advertisement | SB | - | - | 1008 | 30 | 2.98 |
| Lu HY, 2008 | 2006 | Beijing | N | 26.4 | 18-44 | C-S | CDC, peer referral | SB | - | Cell culture | 200 | 1 | 0.50 |
| Wang HL, 2008 | 2006 | Liaoning | NE | - | 18-60 | C-S | - | - | Rectal | Assay Kit | 229 | 0 | 0.00 |
| Wang HL, 2008 | 2006 | Liaoning | NE | - | 18-60 | C-S | - | - | Urethral | Assay Kit | 229 | 0 | 0.00 |
| Wang JX, 2004 | 2003 | Liaoning | NE | - | 17-40 | C-S | Internet advertisement, advertisement, MSM venues | PS | NG-Ab | GIA | 157 | 4 | 2.55 |
| Zhou J, 2008 | 2006 | Guizhou | SW | - | 15-49 | C-S | MSM venues, internet advertisement, telephone hotline | - | - | - | 406 | 6 | 1.48 |
| ***d) Hepatitis B virus*** | | | | | | | | |  | | | | |
| Cai GF, 2008 | 2006 | Zhejiang | E | - | 16-46 | C-S | MSM venues | SB | HBsAg | ELISA | 59 | 6 | 10.17 |
| Chen G, 2010 | 2008 | Fujian | E | - | 18-68 | C-S | MSM venues, peer referral, community outreach | SB, C | HBsAg | ELISA | 159 | 20 | 12.58 |
| Chen SH, 2010 | 2006 | Guangxi | SC | 26.22 | 14-68 | C-S | VCT | C, SB | HBsAg | ELISA | 185 | 24 | 12.97 |
| Chen SH, 2010 | 2007 | Guangxi | SC | 25.47 | 14-68 | C-S | VCT | C, SB | HBsAg | ELISA | 226 | 42 | 18.58 |
| Chen SH, 2010 | 2008 | Guangxi | SC | 27.35 | 14-68 | C-S | VCT | C, SB | HBsAg | ELISA | 905 | 124 | 13.70 |
| Chen SH, 2010 | 2008 | Guangxi | SC | 27.44 | 16-68 | C-S | VCT | SB | HBsAg | ELISA | 452 | 66 | 14.60 |
| Guo H, 2009 | 2006 | Jiangsu | E | - | - | C-S | MSM venues | - | HBsAg | EIA | 296 | 32 | 10.81 |
| Guo H, 2009 | 2007 | Jiangsu | E | - | - | C-S | MSM venues | - | HBsAg | EIA | 137 | 16 | 11.68 |
| Gu Y, 2004 | 2003 | Liaoning | NE | - | 16-49 | C-S | MSM venues | RDS | HBsAg | - | 202 | 11 | 5.45 |
| Han XY, 2007 | 2007 | Shandong | E | - | 17-62 | C-S | VCT | RDS | HBsAg | ELISA | 635 | 48 | 7.56 |
| He Q, 2005 | 2003 | Guangdong | SC | - | 18-55 | C-S | Peer-referral, internet advertisement | SB | HBsAg | ELISA | 117 | 14 | 11.97 |
| He Q, 2006 | 2004 | Guangdong | SC | - | 16-66 | C-S | Internet advertisement, advertisement, peer-referral | C | HBsAg | ELISA | 200 | 35 | 17.50 |
| He Q, 2011 | 2006 | Guangdong | SC | - | 19-62 | C-S | Peer-referral | RDS | HBsAg | ELISA | 423 | 48 | 11.35 |
| Hu JL, 2008 | 2007 | Jiangsu | E | - | 19-54 | C-S | Internet advertisement | PS | HBsAg | ELISA | 65 | 4 | 6.15 |
| Jiang J, 2005 | 2003 | Jiangsu | E | - | 18-70 | C-S | MSM venues | C | HBsAg | EIA | 99 | 9 | 9.09 |
| Lu CG, 2006 | 2005 | Guizhou | SW | - | 15-81 | C-S | MSM venues | SB | HBsAg | - | 276 | 34 | 12.32 |
| Lu CG, 2006 | 2005 | Guizhou | SW | - | 15-81 | C-S | VCT, peer referral, community outreach | SB | HBsAg | - | 200 | 28 | 14.00 |
| Ma X, 2007 | 2004 | Beijing | N | - | - | C-S | Peer-referral | RDS | HBsAg | ELISA | 325 | 28 | 8.62 |
| Ma X, 2007 | 2005 | Beijing | N | - | - | C-S | Peer-referral | RDS | HBsAg | ELISA | 427 | 34 | 7.96 |
| Ma X, 2007 | 2006 | Beijing | N | - | - | C-S | Peer-referral | RDS | HBsAg | ELISA | 540 | 44 | 8.15 |
| Ruan Y, 2009 | 2007 | Beijing | N | - | 18-62 | C-S | Peer-referral, community outreach, internet | - | HBsAg | EIA | 541 | 35 | 6.47 |
| Ruan Y, 2009 | 2007 | Beijing | N | - | 18-62 | CH | Internet advertisement, peer-referral, MSM venues | - | HBsAg | ELISA | 507 | 24 | 4.73 |
| Sun ML, 2009 | 2008 | Liaoning | NE | - | 18-59 | C-S | MSM venues, VCT | PS | HBsAg | ELISA | 401 | 20 | 4.99 |
| Sun ZX, 2007 | - | Zhejiang | E | - | - | C-S | Internet advertisement | - | HBsAg | - | 75 | 4 | 5.33 |
| Wang C, 2012 | 2011 | Beijing | N | - | 16-66 | C-S | Internet advertisement, MSM venues, peer-referral | C | HBsAg | EIA | 1111 | 100 | 9.00 |
| Wang CH, 2007 | 2006 | Hebei | N | - | 19-46 | B-A | MSM venues, internet advertisement | SB | HBsAg | - | 82 | 1 | 1.22 |
| Wang Q, 2009 | 2008 | - | NE | 27.5 | 18-70 | C-S | Community outreach | SB | HBsAg | ELISA | 450 | 38 | 8.44 |
| Xu H, 2009 | - | Liaoning | NE | - | - | C-S | Key person, MSM venues | SB | HBsAg | ELISA | 400 | 26 | 6.50 |
| Xue FH, 2010 | 2009 | Zhejiang | E | 26.96 | 17-49 | C-S | MSM venues | C | HBsAg | ELISA | 203 | 11 | 5.42 |
| Zhang R, 2011 | 2006 | Zhejiang | E | 28.6 | 18-52 | C-S | MSM venues | SB | HBsAg | ELISA | 106 | 12 | 11.32 |
| Zhang R, 2011 | 2007 | Zhejiang | E | 28.6 | 18-52 | C-S | MSM venues | SB | HBsAg | ELISA | 135 | 14 | 10.37 |
| Zhang R, 2011 | 2008 | Zhejiang | E | 28.6 | 18-52 | C-S | MSM venues | SB | HBsAg | ELISA | 177 | 13 | 7.34 |
| Zhang R, 2011 | 2009 | Zhejiang | E | 28.6 | 18-52 | C-S | MSM venues | SB | HBsAg | ELISA | 233 | 16 | 6.87 |
| Zhou J, 2008 | 2006 | Guizhou | SW | - | 15-49 | C-S | MSM venues, internet advertisement, telephone hotline | - | HBsAg | ELISA | 406 | 13 | 3.20 |
| Zhou ZH, 2010 | 2008 | Beijing | N | 27.5 | - | C-S | Community outreach, peer referral, internet advertisement | - | HBsAg | - | 550 | 28 | 5.09 |
| ***e) Hepatitis C virus*** | | | | | | | | |  | | | | |
| Bai Y, 2009 | 2008 | Guangxi | SC | - | - | C-S | Internet advertisement, peer referral, community outreach | - | Anti-HCV | ELISA | 790 | 2 | 0.25 |
| Cai X, 2007 | 2006 | Shandong | E | 26 | 18-38 | C-S | Health education forum | - | Anti-HCV | - | 94 | 2 | 2.13 |
| Chen G, 2010 | 2008 | Fujian | E | - | 18-68 | C-S | MSM venues, peer referral, community outreach | SB, C | IgG | ELISA | 159 | 0 | 0.00 |
| Chen SH, 2010 | 2006 | Guangxi | SC | 26.22 | 14-68 | C-S | VCT | C, SB | Anti-HCV | ELISA | 185 | 2 | 1.08 |
| Chen SH, 2010 | 2007 | Guangxi | SC | 25.47 | 14-68 | C-S | VCT | C, SB | Anti-HCV | ELISA | 226 | 0 | 0.00 |
| Chen SH, 2010 | 2008 | Guangxi | SC | 27.35 | 14-68 | C-S | VCT | C, SB | Anti-HCV | ELISA | 905 | 5 | 0.55 |
| Chen SH, 2010 | 2008 | Guangxi | SC | 27.44 | 16-68 | C-S | VCT | SB | Anti-HCV | ELISA | 452 | 3 | 0.66 |
| Chen Z, 2011 | 2010 | Yunnan | SW | 27.3 | 15-67 | C-S | MSM venues | SB | Anti-HCV | ELISA | 200 | 2 | 1.00 |
| Ding XB, 2010 | 2008 | Chongqing | SW | 26.3 | 18-67 | C-S | VCT, community outreach | SB | Anti-HCV | ELISA | 743 | 27 | 3.63 |
| Feng F, 2009 | 2008 | Hainan | SC | 28.3 | 18-55 | C-S | MSM venues, peer referral, internet advertisement | SB | Anti-HCV | - | 104 | 0 | 0.00 |
| Feng JF, 2008 | 2007 | Zhejiang | E | - | - | C-S | MSM venues | C | Anti-HCV | ELISA | 73 | 0 | 0.00 |
| Feng LG, 2010 | 2008 | Chongqing | SW | - | - | C-S | Internet advertisement, MSM venues | SB | Anti-HCV | ELISA | 945 | 45 | 4.76 |
| Gao LM, 2010 | 2010 | Yunnan | SW | 33.3 | - | C-S | MSM venues, community outreach | - | Anti-HCV | - | 105 | 3 | 2.86 |
| Gong CT, 2010 | 2009 | Fujian | E | 22 | 19-46 | C-S | MSM venues | SB | Anti-HCV | RIBA | 229 | 0 | 0.00 |
| Guo H, 2009 | 2006 | Jiangsu | E | - | - | C-S | MSM venues | - | Anti-HCV | EIA | 296 | 3 | 1.01 |
| Guo H, 2009 | 2007 | Jiangsu | E | - | - | C-S | MSM venues | - | Anti-HCV | EIA | 137 | 0 | 0.00 |
| Gu Y, 2004 | 2003 | Liaoning | NE | - | 16-49 | C-S | MSM venues | RDS | Anti-HCV | - | 202 | 5 | 2.48 |
| Han X, 2011 | 2010 | Qinghai | NW | 27.7 | 18-55 | C-S | MSM venues | SB | Anti-HCV | ELISA | 312 | 2 | 0.64 |
| Han XY, 2007 | 2007 | Shandong | E | - | 17-62 | C-S | VCT | RDS | Anti-HCV | ELISA | 635 | 2 | 0.31 |
| He H, 2011 | 2010 | Hunan | SC | 26 | 16-59 | C-S | HIV/AIDS education programs | - | Anti-HCV | - | 117 | 0 | 0.00 |
| He Q, 2005 | 2003 | Guangdong | SC | - | 18-55 | C-S | Peer-referral, internet advertisement | SB | Anti-HCV | ELISA | 117 | 0 | 0.00 |
| He Q, 2006 | 2004 | Guangdong | SC | - | 16-66 | C-S | Internet, advertisement, peer referral | C | Anti-HCV | ELISA | 200 | 2 | 1.00 |
| He Q, 2011 | 2006 | Guangdong | SC | - | 19-62 | C-S | Peer-referral | RDS | Anti-HCV | ELISA | 423 | 12 | 2.84 |
| Hu JF, 2011 | 2010 | Jiangxi | E | 22 | 16-45 | C-S | Community outreach, peer referral | - | Anti-HCV | ELISA | 106 | 2 | 1.89 |
| Hu JL, 2008 | 2007 | Jiangsu | E | - | 19-54 | C-S | Internet advertisement | PS | Anti-HCV | ELISA | 65 | 1 | 1.54 |
| Huang HY, 2011 | 2008 | Anhui | E | 27.6 | 18-72 | C-S | MSM venues | SB | Anti-HCV | EIA | 395 | 4 | 1.01 |
| Jiang J, 2005 | 2003 | Jiangsu | E | - | 18-70 | C-S | MSM venues | C | Anti-HCV | EIA | 93 | 0 | 0.00 |
| Lan GH, 2009 | 2008 | Guangxi | SC | 30.06 | 18-59 | C-S | - | - | Anti-HCV | ELISA | 179 | 0 | 0.00 |
| Liang L, 2009 | 2008 | Hebei | N | - | 18-49 | C-S | VCT, peer referral | SB | Anti-HCV | - | 450 | 2 | 0.44 |
| Liu PL, 2010 | 2008 | Hubei | SC | - | - | C-S | Community outreach, MSM venues | RDS | Anti-HCV | ELISA | 456 | 10 | 2.19 |
| Liu YY, 2010 | 2009 | Hunan | SC | 25.84 | 16-65 | C-S | MSM venues, internet | SB | Anti-HCV | RIBA | 507 | 3 | 0.59 |
| Lu CG, 2006 | 2005 | Guizhou | SW | - | 15-81 | C-S | MSM venues | SB | Anti-HCV | - | 276 | 12 | 4.35 |
| Lu CG, 2006 | 2005 | Guizhou | SW | - | 15-81 | C-S | VCT, peer referral, community outreach | SB | Anti-HCV | - | 200 | 18 | 9.00 |
| Lu L, 2011 | 2009 | Jiangxi | E | 26.2 | 19-58 | C-S | MSM Venues | SB | Anti-HCV | RIBA | 354 | 2 | 0.56 |
| Ma GL, 2010 | 2008 | Fujian | E | 25.31 | - | B-A | Community outreach, internet advertisement, peer referral, MSM venues | SB | Anti-HCV | - | 98 | 1 | 1.02 |
| Ma GL, 2010 | 2008 | Fujian | E | 26.21 | - | B-A | Community outreach, internet advertisement, peer referral, MSM venues | SB | Anti-HCV | - | 140 | 1 | 0.71 |
| Ma GL, 2010 | 2009 | Fujian | E | 25.58 | - | B-A | Community outreach, internet advertisement, peer referral, MSM venues | SB | Anti-HCV | - | 154 | 1 | 0.65 |
| Ma X, 2007 | 2004 | Beijing | N | - | - | C-S | Peer-referral | RDS | Anti-HCV | ELISA | 325 | 3 | 0.92 |
| Ma X, 2007 | 2005 | Beijing | N | - | - | C-S | Peer-referral | RDS | Anti-HCV | ELISA | 427 | 7 | 1.64 |
| Ma X, 2007 | 2006 | Beijing | N | - | - | C-S | Peer-referral | RDS | Anti-HCV | ELISA | 540 | 23 | 4.26 |
| Mao FY, 2010 | 2010 | Hubei | SC | 28.17 | 16-58 | C-S | Community outreach, group counselling | SB, Time-location-based sampling | Anti-HCV | ELISA | 211 | 0 | 0.00 |
| Mei L, 2009 | 2008 | Shanxi | N | 24 | 18-49 | C-S | MSM venues, VCT | SB | Anti-HCV | ELISA | 273 | 2 | 0.73 |
| Meng X, 2010 | 2009 | Hunnan | SC | - | 18-73 | C-S | VCT, peer referral, community outreach, internet | C | Anti-HCV | ELISA | 287 | 3 | 1.05 |
| Miao XL, 2011 | 2010 | Jiangsu | E | - | - | C-S | MSM venues | Continuous | Anti-HCV | ELISA | 259 | 3 | 1.16 |
| Miao ZF, 2009 | 2008 | Ningxia | NW | 27.7 | 18-55 | C-S | MSM venue | SB | Anti-HCV | - | 312 | 2 | 0.64 |
| Nie ZQ, 2011 | 2009 | Guangdong | E | - | 19-51 | C-S | MSM venues, internet advertisement | Stratified Proportional Sampling | Anti-HCV | ELISA | 570 | 3 | 0.53 |
| Ruan Y, 2009 | 2007 | Beijing | N | - | 18-62 | C-S | Peer-referral, community outreach, internet advertisement | - | Anti-HCV | EIA | 541 | 2 | 0.37 |
| Ruan Y, 2009 | 2007 | Beijing | N | - | 18-62 | CH | Internet advertisement, peer-referral, MSM venues | - | Anti-HCV | ELISA | 507 | 1 | 0.20 |
| Shi JC, 2010 | 2009 | Henan | SC | 32 | 17-66 | C-S | Internet advertisement, telephone hotline, peer referral | - | Anti-HCV | - | 138 | 3 | 2.17 |
| Shi WD, 2009 | 2008 | Hubei | SC | 28.27 | 18-60 | C-S | VCT | RDS | Anti-HCV | ELISA | 456 | 11 | 2.41 |
| Sun DY, 2010 | 2009 | Henan | SC | - | - | C-S | Community outreach, MSM venues | RDS | Anti-HCV | ELISA | 1350 | 80 | 5.93 |
| Sun ML, 2009 | 2008 | Liaoning | NE |  | 18-59 | C-S | MSM venues, VCT | PS | Anti-HCV | ELISA | 401 | 7 | 1.75 |
| Wang C, 2008 | 2007 | Beijing | N | 28.2 | - | C-S | VCT, community outreach, peer referral | - | Anti-HCV | - | 541 | 1 | 0.18 |
| Wang C, 2012 | 2011 | Beijing | N | - | 16-66 | C-S | Internet advertisement, MSM venues, peer-referral | C | Anti-HCV | ELISA | 1111 | 43 | 3.87 |
| Wang CH, 2007 | 2006 | Hebei | N | - | 19-46 | B-A | MSM venues, internet advertisement | SB | Anti-HCV | - | 82 | 0 | 0.00 |
| Wang Q, 2009 | 2008 |  | NE | 27.5 | 18-70 | C-S | Community outreach | SB | Anti-HCV | ELISA | 450 | 11 | 2.44 |
| Wang T, 2010 | 2009 | Guangdong | E | 26 | 17-52 | C-S | VCT | - | Anti-HCV | ELISA | 207 | 0 | 0.00 |
| Wang X, 2011 | 2009 | Xinjiang | NW | 24.62 | - | C-S | MSM venues | SB | Anti-HCV | ELISA | 201 | 5 | 2.49 |
| Wang Y, 2009 | 2007 | Sichuan | SW | 24 | 16-40 | C-S | - | RDS | Anti-HCV | ELISA | 111 | 1 | 0.90 |
| Wang ZJ, 2010 | 2009 | Jiangsu | E | 33.47 | 18-78 | C-S | MSM venues, internet advertisement | - | Anti-HCV | - | 750 | 2 | 0.27 |
| Weng YQ, 2009 | - | Guangxi | SC | 27.88 | 18-57 | C-S | Peer-referral, community outreach, VCT | SB | Anti-HCV | ELISA | 239 | 0 | 0.00 |
| Wu J, 2011 | 2010 | - | - | 29.3 | 18-67 | C-S | MSM venues | SB | Anti-HCV | ELISA | 430 | 6 | 1.40 |
| Xu H, 2009 | - | Liaoning | NE | - | - | C-S | Key person, MSM venues | SB | Anti-HCV | ELISA | 400 | 4 | 1.00 |
| Xu XY, 2011 | 2009 | Inner Mongolia | N | 32 | 17-84 | C-S | MSM venues | - | Anti-HCV | ELISA | 401 | 9 | 2.24 |
| Zhang DD, 2010 | 2009 | Zhejiang | E | 28.1 | 18-67 | C-S | MSM venues | SB | Anti-HCV | ELISA | 520 | 14 | 2.69 |
| Zhang H, 2011 | 2010 | Fujian | E | 29 | 16-64 | C-S | MSM venues | Continuous | Anti-HCV | ELISA | 147 | 3 | 2.04 |
| Zhang M, 2009 | 2008 | Xinjiang | NW | - | - | C-S | Peer-referral | RDS | Anti-HCV | ELISA | 231 | 3 | 1.30 |
| Zhang R, 2011 | 2006 | Zhejiang | E | 28.6 | 18-52 | C-S | MSM venues | SB | Anti-HCV | ELISA | 106 | 0 | 0.00 |
| Zhang R, 2011 | 2007 | Zhejiang | E | 28.6 | 18-52 | C-S | MSM venues | SB | Anti-HCV | ELISA | 135 | 0 | 0.00 |
| Zhang R, 2011 | 2008 | Zhejiang | E | 28.6 | 18-52 | C-S | MSM venues | SB | Anti-HCV | ELISA | 177 | 1 | 0.56 |
| Zhang R, 2011 | 2009 | Zhejiang | E | 28.6 | 18-52 | C-S | MSM venues | SB | Anti-HCV | ELISA | 233 | 0 | 0.00 |
| Zhang X, 2007 | 2006 | Beijing | N | 26.1 | 18-55 | C-S | VCT | PS | Anti-HCV | ELISA | 753 | 6 | 0.80 |
| Zhang XY, 2006 | 2005 | Beijing | N | - | - | C-S | - | - | Anti-HCV | ELISA | 526 | 8 | 1.52 |
| Zhang Y, 2008 | 2007 | Xinjiang | NW | - | - | C-S | Internet advertisement | - | Anti-HCV | ELISA | 143 | 0 | 0.00 |
| Zhang ZK, 2010 | 2008 | Guangxi | SC | 28.28 | - | C-S | MSM venues | - | Anti-HCV | ELISA | 315 | 1 | 0.32 |
| Zhao XH, 2010 | 2009 | Sichuan | SW | 25 | 11-76 | C-S | MSM venues, internet advertisement | - | Anti-HCV | - | 362 | 1 | 0.28 |
| Zhou C, 2011 | 2008 | Chongqing | SW | 25.8 | 18-67 | C-S | CDC | SB | Anti-HCV | - | 1166 | 29 | 2.49 |
| Zhou CX, 2011 | 2010 | Guizhou | SW | 24.78 | 14-52 | C-S | VCT | - | Anti-HCV | - | 102 | 1 | 0.98 |
| Zhou CX, 2010 | - | Guizhou | SW | 25.1 | 18-51 | C-S | VCT | SB | Anti-HCV | - | 170 | 3 | 1.76 |
| Zhou J, 2008 | 2006 | Guizhou | SW |  | 15-49 | C-S | MSM venues, internet advertisement, telephone hotline | - | Anti-HCV | ELISA | 406 | 8 | 1.97 |
| Zhou ZH, 2010 | 2008 | Beijing | N | 27.5 | - | C-S | Community outreach, peer referral, internet advertisement | - | Anti-HCV | - | 550 | 3 | 0.55 |
| ***f) HPV*** | | | | | | | | |  | | | | |
| Chen XX, 2011 | 2010 | Beijing | N | - | - | C-S | NGOs | - | Any type | PCR | 283 | 200 | 70.67 |
| Chen XX, 2011 | 2010 | Beijing | N | - | - | C-S | NGOs | - | HPV16 | PCR | 283 | 13 | 4.59 |
| Chen XX, 2011 | 2010 | Beijing | N | - | - | C-S | NGOs | - | HPV06 | PCR | 283 | 20 | 7.07 |
| Chen XX, 2011 | 2010 | Beijing | N | - | - | C-S | NGOs | - | HPV45 | PCR | 283 | 9 | 3.18 |
| Chen XX, 2011 | 2010 | Beijing | N | - | - | C-S | NGOs | - | Multiple types | PCR | 283 | 108 | 38.16 |
| Chen XX, 2011 | 2010 | Beijing | N | - | - | C-S | NGOs | - | Single type | PCR | 283 | 92 | 32.51 |
| Gao L, 2010 | 2010 | Beijing and Tianjin | N | - | - | C-S | Internet advertisement, distributing flyers with study-related information at MSM venues | - | Any type | PCR | 578 | 359 | 62.11 |
| Gao L, 2012 | 2010 | Beijing and Tianjin | N | - | - | C-S | Internet advertisement, distributing flyers with study-related information at MSM venues | - | HPV16 | PCR | 578 | 75 | 12.98 |
| Gao L, 2012 | 2010 | Beijing and Tianjin | N | - | - | C-S | Internet advertisement, distributing flyers with study-related information at MSM venues | - | HPV18 | PCR | 578 | 34 | 5.88 |
| Gao L, 2012 | 2010 | Beijing and Tianjin | N | - | - | C-S | Internet advertisement, distributing flyers with study-related information at MSM venues | - | HPV31 | PCR | 578 | 0 | 0.00 |
| Gao L, 2012 | 2010 | Beijing and Tianjin | N | - | - | C-S | Internet advertisement, distributing flyers with study-related information at MSM venues | - | HPV45 | PCR | 578 | 31 | 5.36 |
| Gao L, 2012 | 2010 | Beijing and Tianjin | N | - | - | C-S | Internet advertisement, distributing flyers with study-related information at MSM venues | - | Multiple types | PCR | 578 | 169 | 29.24 |
| Gao L, 2012 | 2010 | Beijing and Tianjin | N | - | - | C-S | Internet advertisement, distributing flyers with study-related information at MSM venues | - | Single type | PCR | 578 | 190 | 32.87 |
| ***f) Herpes Simplex Virus Type 2*** | | | | | | | | |  | | | | |
| Cai WD, 2005 | 2004 | Guangdong | SC | 25.9 | 17-19 | C-S | MSM venues | PS | - | - | 261 | 43 | 16.48 |
| Ding XB, 2010 | 2008 | Chongqing | SW | 26.3 | 18-67 | C-S | VCT, community outreach | SB | IgG | Assay Kit | 743 | 25 | 3.36 |
| Feng Y, 2010 | 2007 | Sichuan | SW | - | - | C-S | MSM venues | SB | IgG | ELISA | 538 | 133 | 24.72 |
| Jiang J, 2005 | 2003 | Jiangsu | E | - | 18-70 | C-S | MSM venues | C | IgG | ELISA | 90 | 7 | 7.78 |
| Yin YP, 2012 | 2010 | Guangdong, Jiangsu |  | 30.14 | 18-66 | C-S | STD clinic, health center, MSM venues | - | IgG | ELISA | 1462 | 234 | 16.01 |
| Zheng M, 2011 | 2009 | Guizhou | SW | 25.12 | 18-52 | C-S | Peer-referral, Internet advertisement | SB | - | ELISA | 357 | 22 | 6.16 |
| ***h) Ureaplasma urealyticum*** | | | | | | | | |  | | | | |
| Chen SH, 2007 | 2006 | Guangxi | SC | 26.22 | 18-45 | C-S | VCT, peer-referral, internet advertisement | - | Urethral swab | Cell culture | 185 | 7 | 3.78 |
| Zhang X, 2007 | 2006 | Beijing | N | 26.1 | 18-55 | C-S | VCT | PS | IgM/IgG | Assay Kit | 753 | 33 | 4.38 |

^Anti-HCV: detects the presence of antibodies to the hepatitis C virus.

^#^­Six Chinese traditional regions. E: East (Anhui, Fujian, Jiangsu, Jiangxi, Shandong, Shanghai, and Zhejiang); N: North (Beijing, Hebei, Inner Mongolia, Shanxi, and Tianjin); NE: Northeast (Heilongjiang, Jilin, and Liaoning; North China); NW: Northwest (Gansu, Ningxia, Qinghai, Shaanxi, and Xinjiang); SC: South Central (Guangdong, Guangxi, Hainan, Henan, Hubei, and Hunan); SW: Southwest (Chongqing, Guizhou, Sichuan, Tibet, and Yunnan).

^ϕ^B-A: Before-and-After study; CH: Cohort study; C-S: Cross-sectional study.

^φ^ CDC: recruited by Chinese Center for Disease Control and Prevention; MSM venues: gay-oriented venues such as gay bars, saunas, bathhouse, and clubs; NGOs: recruited by non-governmental organisations. VCT: HIV voluntary counselling and testing sites;

^δ^ C: Convenience; RDS: Respondent-driven sampling; SB: Snowball; PS: Pilot survey

*ELISA: Enzyme-linked immunosorbent assay; IgG: Immunoglobulin G; IgM: Immunoglobulin M; PCR: The polymerase chain reaction; GIA: Colloidal Gold Immunoassay; EIA: Enzyme Immunoassay.

**References**

1. Chen SH, Zhu JQ, Yang NH: **[Investigation on HIV and STI Infections among Men Who Have Sex with Men in Nanning City during 2006-2008]**. *Occupation and Health* 2010, **26**(1):56-58.

2. Chen SH, Zhu JJ, Li J: **[Investigation on knowledge of HIV/AIDS and related behaviors character among men who have sex with men in Nanning, Guangxi]**. *Chinese Journal of Disease Control & Prevention* 2010, **14**(2):130-133.

3. Chen XM, Fu G, Xu X: **[Study on gonococcal and chlamydial infections among men who have sex with men in Nanjing]**. *Acta Universitatis Medicinalis Anhui* 2011, **46**(6):569-572.

4. Huan XP, Yun YP, Fu GF, Jiang N, Zhang QQ, Zhang XN, Wang XL, Hu HY, Wang B, Yang HT *et al*: **[Analysis on sexually transmitted diseases and the related risk factors among men who have sex with men in Jiangsu province]**. *Chinese Journal of Preventive Medicine* 2011, **45**(11):975-978.

5. Jiang J, Cao N, Zhang J, Xia Q, Gong X, Xue H, Yang H, Zhang G, Shao C: **High prevalence of sexually transmitted diseases among men who have sex with men in Jiangsu Province, China**. *Sex Transm Dis* 2006, **33**(2):118-123.

6. Lu HY, Ma XY, Liu YC, Zhang QY, Hei FX, Zhang Q, He X, Wang N: **[A survey of HIV/STDs prevalence in 200 MSM and related factors in Beijing]**. *Chinese Journal of AIDS & STD* 2008, **14**(5):467-470.

7. Wang HL, Zhang M, Hu QH, Ding HB, Zhao B: **[Prevalence of HIV/STD and risk behavior among men who have sex with men in Shenyang]**. *Chinese Journal of Public Health* 2008, **24**(8):995-997.

8. Zhang X, Wang C, Hengwei W, Li X, Li D, Ruan Y, Shao Y: **Risk factors of HIV infection and prevalence of co-infections among men who have sex with men in Beijing, China**. *AIDS* 2007, **21 Suppl 8**:S53-57.

9. Gao LM, Chen L, Ma Y, Lu JB, Li LX, Zhang YL: **[HIV/STD infection and KABP status among men who have sex with men in Yuxi city, Yunnan province, 2009-2010]**. *Soft Science of Health* 2010, **24**(6):547-549, 553.

10. Zhou J, Zhu JJ, Bin H, Zhang L, Yao M, Zhang W, Zhang W, Zhong JY, You QY, Gao L: **[A survey of HIV/STD, HBV and HCV infections and risk behaviors among MSM in two central districts of Guiyang city]**. *Chinese Journal of AIDS & STD* 2008, **14**(1):47-48, 51.

11. Liu Y, Jiang S, Hu Y, SOng L, Yu M, Li SM: **[Characteristics of sexual behaviors and infection status of AIDS and other sexually transmitted diseases among men who have sex with men in 2009 in Beijing]**. *Zhonghua Yu Fang Yi Xue Za Zhi* 2011, **45**(11):971-974.

12. Wang JX, Dong W, Li R, Zhao Z, Xiong B, Gang J: **[A survey of HIV/STD infection and behaviors among MSM]**. *Chinese Journal of Public Health* 2004, **20**(11):1377-1378.

13. Cai GF, Ma QQ, Pan XH, Fu LJ, Xu WX, Shan XR, Yang Q: **[HIV/AIDS Related Knowledge, Attitude, Practice and HIV/STD Infection among MSM in Two Cities of Zhejiang Province]**. *China Preventive Medicine* 2008, **9**(6):482-485.

14. Chen G, Zheng JQ, Pan YJ, Zhang CY, Lin L: **[Serological Surveillance and Analysis among Men Having Sex with Men in a City of Fujian, China]**. *Strait Journal of Preventive Medicine* 2010, **16**(2):13-16.

15. Guo H, Wei JF, Yang H, Huan X, Tsui SK, Zhang C: **Rapidly increasing prevalence of HIV and syphilis and HIV-1 subtype characterization among men who have sex with men in Jiangsu, China**. *Sex Transm Dis* 2009, **36**(2):120-125.

16. Gu Y, Qu P, Xu L, Luo M, Wang X, Gu J, Zhao L, Lu Y-h, Zhou B: **[Survey of knowledge, attitude, behavior and practice related to STI/HIV among male homosexuality in Shenyang]**. *Chinese Journal of Public Health* 2004, **20**(5):573-574.

17. Han XY, Liu LZ, Zhang W, Ma DN, Chu Q, Chu FL, Pan J: **[Investigation on the Related Nosogenic Infection among 635 Men Who Have Sex with Men in Ji'nan]**. *Preventive Medicine Tribune* 2007, **13**(12):1091-1092.

18. He Q, Wang Y, Lin P, Zhang Z-b, Zhao X-x, Xu H-f: **[KAP study on AIDS among men who have sex with men in Guangzhou, Guangdong province]**. *Chinese Journal of Disease Control & Prevention* 2005, **9**(2):106-108.

19. He Q, Wang Y, Lin P, Liu Y, Yang F, Fu X, Li Y, Sun B, Li J, Zhao X *et al*: **Potential bridges for HIV infection to men who have sex with men in Guangzhou, China**. *AIDS Behav* 2006, **10**(4 Suppl):S17-23.

20. He Q, Wang Y, Lin P, Raymond HF, Li Y, Yang F, Zhao J, Li J, Ling L, McFarland W: **High prevalence of risk behaviour concurrent with links to other high-risk populations: a potentially explosive HIV epidemic among men who have sex with men in Guangzhou, China**. *Sex Transm Infect* 2009, **85**(5):383-390.

21. Hu JL, Wu LP, Zhang ZH, Fan W: **[Investigation of STD/AIDS infection, related knowledge and high-risk sexual behavior among MSM in Huaian city]**. *Journal of Public Health and Preventive Medicine* 2008, **19**(5):44-45.

22. Lu CG, Yuan F, Shi Z, Yang JZ, Li XY, Gao L, Li X, Hu SY: **[The study of HIV infection and KABP about AIDS among the MSM in Guiyang city]**. *Guizhou Medical Journal* 2006, **30**(3):202-204.

23. Lu CG, Yuan F, Shi ZH, Yang JZ, Li XY, Gao L, Lu X, Hu SY: **[HIV infection survey among MSM]**. *Chinese Journal of Public Health* 2006, **2**(11):1320-1321.

24. Ma X, Zhang Q, He X, Sun W, Yue H, Chen S, Raymond HF, Li Y, Xu M, Du H *et al*: **Trends in Prevalence of HIV, Syphilis, Hepatitis C, Hepatitis B, and Sexual Risk Behavior Among Men Who Have Sex With Men. Results of 3 consecutive respondent-driven sampling surveys in Beijing, 2004 through 2006**. *J Acquir Immune Defic Syndr* 2007, **45**(5):581-587.

25. Ruan Y, Luo F, Jia Y, Li X, Li Q, Liang H, Zhang X, Li D, Shi W, Freeman JM *et al*: **Risk factors for syphilis and prevalence of HIV, hepatitis B and C among men who have sex with men in Beijing, China: implications for HIV prevention**. *AIDS Behav* 2009, **13**(4):663-670.

26. Ruan Y, Jia Y, Zhang X, Liang H, Li Q, Yang Y, Li D, Zhou Z, Luo F, Shi W *et al*: **Incidence of HIV-1, syphilis, hepatitis B, and hepatitis C virus infections and predictors associated with retention in a 12-month follow-up study among men who have sex with men in Beijing, China**. *J Acquir Immune Defic Syndr* 2009, **52**(5):604-610.

27. Sun ML, Li DJ, Jin W, Jiang J, Guan L: **[Investigation on the Infection of HIV, HCV, Syphilis and HBV among MSM in Dalian City in 2008]**. *Preventive Medicine Tribune* 2009, **15**(11):1074 - 1075.

28. Sun ZX, Lin SF, Wen MQ: **[Investigation on STD and AIDS prevalence for men who have sex with men]** *Modern Preventive Medicine* 2007, **34**(21):4130-4132.

29. Wang C, Wang Y, Huang X, Li X, Zhang T, Song M, Wu L, Du J, Lu X, Shao S *et al*: **Prevalence and Factors Associated with Hepatitis B Immunization and Infection among Men Who Have Sex with Men in Beijing, China**. *PLoS One* 2012, **7**(10):e48219.

30. Wang CH, Yang YH, Lu GJ, Yang XX, Ge LR, Zhao HR, Zhang YQ: **[Intervent male male sexual contact AIDS high dangerous behavior and evaluate effection of intervention]**. *Chinese Journal of Health Laboratory Technology* 2007, **17**(12):2291-2292.

31. Wang Q, Hou Y, Lian YZ: **[Investigation on the Infection of HIV and Sex Transmitted Diseases in Male Homosexual Population]**. *Strait Journal of Preventive Medicine* 2009, **15**(3):11-12.

32. Xu H, Hu W, Zhao HY: **[AIDS survey of MSM homosexuality population in Anshan]**. *Chinese Journal of Health Laboratory Technology* 2009, **19**(3):658,713.

33. Xue FH, Xia SZ, Lin SF, Jin YH, Zhu HS, Wen MQ: **[Survey on high risk behaviors and HIV/STD infection status among men who have sex with men]**. *Disease Surveillance* 2010, **25**(1):54-56.

34. Zhang R, Shuai HQ, Shang XC: **[Investigation on the infection of HIV and Sex Transmitted Diseases in the man who have sex with man from 2006 to 2009 in Hangzhou]**. *Chinese Journal of Health Laboratory Technology* 2011, **21**(3):732-733.

35. Zhou ZH, Li SM, Liu YJ, Li QC, Li DL, Zhang Z, Jiang SL, Luo FJ, Shi W, Ruan YH *et al*: **[Survey of HIV and syphilis infection and influential factors assoicated with unprotected anal intercourse in men who have sex with men in Beijing]**. *China Tropical Medicine* 2010, **10**(1):10-12.

36. Bai Y, Feng WD, Wei QH: **[Survey of infectious status of HIV,HCV and syphilis in MSM in Liuzhou in 2007-2008]**. *China Tropical Medicine* 2009, **9**(12):2275-2276.

37. Cai X: **[The Analysis of Risk Behaviors of MSM in Liaocheng and the Serological Detection for Anti-HIV, Anti-TP, and Anti-HCV Antibodies in 2006]**. *Preventive Medicine Tribune* 2007, **13**(10):888-890.

38. Chen Z, Chen XM, Huang LH, Lu MJ, You YT, Xiong CM, Li JR: **[Sentinel surveillance among men who have sex with men in Dali prefecture of Yunnan province in 2010]**. *Soft Science of Health* 2011, **25**(10):719-720, 728.

39. Ding XB, Feng LG, Xu J, Xu SM, Guo Xj, Zheng JQ, Yang MF, Liu XP: **[Study on the prevalence of HIV, Syphilis, HCV and HSV-II and its associated factors among 743 men who have sex with men in Chongqing]**. *Chinese Journal of Disease Control & Prevention* 2010, **14**(3):227-231.

40. Feng F, Wang ZQ, Huang SP, Lu JG, Lin ZW, Zhong X: **[Investigation on aids knowledge, attitude and practice characteristics of MSM group and HIV/syphilis infection situation]**. *Modern Preventive Medicine* 2009, **36**(15):2902-2903, 2909.

41. Feng JF, Lin HJ, Qiu DH, Zhang YF, Wu QH: **[Study on infection rate of HIV,syphilis and HCV among men who have sex with men in Taizhou]**. *Chinese Journal of Health Laboratory Technology* 2008, **18**(9):1885-1886.

42. Feng LG, Ding XB, Xu j, Ou YL, Xu SM, Zheng JQ, Guo XJ, Yang MF, Liu XP: **[Study on HIV, Syphilis and HCV Prevalence and Its Assoicated Factors among Internet MSM Comparison to Non-Internet MSM in Chongqing]**. *Journal of Tropical Medicine* 2010, **10**(1):78-82.

43. Gong CT, Zhang QH: **[Results of AIDS monitoring of 252 MSM in Quanzhou City]**. *China Tropical Medicine* 2010, **10**(12):1496-1497.

44. Han X, Zhang XP, Li J, Lei LM, Miao ZF: **[Investigation on high-risk behaviour and Sexually transmissible infections among men who have sex with men in Yinchuan city of Ningxia province in 2010]**. *Ningxia Medical Journal* 2012, **34**(1):69-70.

45. He H, Zhang D, Zhou L, Ye P, Bai Z, Zhu L, Chen H, Li Z: **[Behavioral Characters & Practice of Behavior Intervention Among 135 MSM in Yueyang]**. *Practical Preventive Medicine* 2011, **18**(6):1151-1153.

46. Hu JF, Wang YN, Li J: **[Study on HIV, syphilis and HCV infection and high risk behaviors among men who have sex with men in Donghu, Jiangxi Province]**. *World Health Digest* 2011, **8**(17):68-69.

47. Huang HY, Zhang XP, Li Y, Hu ZW, Li ZR: **[Study on the HIV, syphilis and hepatitis C infections and sexual behavior characteristics among men who have sex with men in Hefei]**. *Modern Preventive Medicine* 2011, **38**(10):1933-1935, 1938.

48. Lan GH, Liu W, Zhu QY, Liang FX, Zhou Y: **[To analyze the surveillance results of immigrant MSM in Guangxi]**. *Applied Prev Med* 2009, **15**(3):168-170.

49. Liang L, Chen ZQ, Miao XF, Li Bj, Bai GY, Zhao HR: **[An investigation of HIV infections among men who have sex with men]**. *Hebei Medical Journal* 2009, **31**(15):1991-1992.

50. Liu PL, Yao ZZ, Shi WD, Ding J, Li SL, Chen L, Yang T, Wang X, Zhou W: **[Epidemiological study on the status of HIV/STD among MSM in Wuhan City]**. *Chinese Journal of Disease Control and Prevention* 2010, **14**(9):917-919.

51. Liu YY, Tian B, Song WW: **[Study on the prevalence of HIV, HCV and syphilis and demographic characteristics among 507 men who have sex with men]**. *Practical Preventive Medicine* 2010, **17**(4):788-790.

52. Lu L, Xu D, Qiu HH, Yan H, Chen RC, Cao Y, Li SY: **[Investigation on STDs /AIDS infection amongMSM in Nanchang]**. *Journal of Public Health and Preventive Medicine* 2011, **22**(1):45-46.

53. Ma GL, Shen LT, Su CH, Zheng HN, Lin BY, Li L: **[Effect evaluation of HIV/AIDS integrated intervention of MSM in Xiamen City]**. *Chinese Journal of Disease Control and Prevention* 2010, **14**(8):726-728.

54. Mao FY: **[Survey on HIV/STI high risk behaviors among 211 men who have sex with men in Yichang city, Huebi province]**. *World Health Digest* 2010, **7**(31):246-247.

55. Mei L, Han h, Che XW, Bai SM, Yang YL, Jie ZL, Wang JT, Yuan CL: **[A survey of the first round AIDS integrate controlling work trial spot among men who have sex with man in Taiyuan]**. *Chinese Journal of Epidemiology* 2009, **30**(6):649-650.

56. Meng X, Xie CM, Zheng XP: **[Investigation on AIDS and STD Affection in MSM of Changde]**. *Journal of Tropical Medicine* 2010, **10**(7):884-885.

57. Miao XL, Cheng H, Zhang X, Gu J, Ji YY, He EQ: **[Analysis on HIV/AIDS Sentinel Surveillance in Wuxi City in 2010]**. *Occupation and Health* 2011, **27**(22):2599-2601.

58. Miao ZF, Li J, Lei LM, Han X, Zhang XP: **[Survey of AIDS-related Knowledge and Behavior in 312 MSM]**. *Journal of Ningxia Medical University* 2009, **31**(6):761-762.

59. Nie ZQ, Lin P, Li Y, Wang Y: **[Surveillance of AIDS high-risk people in Guangdong province, 2009]**. *J Trop Med* 2011, **11**(1):29-31, 45.

60. Shi JC, Lu QC, Zhao T: **[Analysis of the knowledge and infection on AIDS among the MSM in Nanyang City]**. *Henan J Prev Med* 2010, **21**(6):405-406, 416.

61. Shi WD, Li G, Yang T, Zhou W, Liu PL, Li SL, Chen L, Wei J: **[Survey of High Risk Sexual Behaviors and HIV,Syphilis,HCV among 456 Male Homosexuals in Wuhan]**. *Medicine and Society* 2009, **22**(10):42-43.

62. Sun DY, Ma YM, Nie YG, Zhu Q, Wang Z: **[Study on HIV/HCV and syphilis infection among MSM in two cities]**. *Chinese journal of Practical Medicine* 2010, **37**(21):10-12.

63. Wang C, Liang HY, Yang Y, Zeng ZL, Li QC: **[A survey of HIV infections and related factors among men who have sex with men in Beijing]**. *Chinese Journal of AIDS & STD* 2008, **14**(6):552-557.

64. Wang T, Lai XH, Li L, Chen CY, He BH: **[Survey on AIDS/STD Risk Behaviors and Prevalence Among Men Who Have Sex with Men in Zhongshan, Guangdong]**. *Practical Preventive Medicine* 2010, **17**(7):1261-1263.

65. Wang X, Ma Y, Yang Y: **[Prevalence of HIV, syphilis and HCV among men who have sex with men in Urumqi city of Xinjiang Uyghur Autonomous Region]**. *Journal of Dermatology and Venereology* 2011, **33**(2):104-105.

66. Wang Y, Zhang HB, Zhang GG, Yang HW, Fan J, Jia SG: **[Biological Monitoring and Social Characteristics Investigation of MSM Group in Mianyang City]**. *Practical Preventive Medicine* 2009, **16**(2):375-377.

67. Wang ZJ, Sun L, Ma XJ: **[Survey on AIDS/STD risk behaviors and prevalence among men who have sex with men in Guangling,Yangzhou]**. *Jiangsu Journal of Preventive Medicine* 2010, **21**(2):4-7.

68. Weng YQ, Bai Y: **[Surveillance on the high risk behaviors among 239 men who have sex with men]**. *Journal of Applied Preventive Medicine* 2009, **15**(3):152-153.

69. Wu J, Ning Z, Fan HL, Yuan J, Lin: **[Sentinel surveillance of sexually transmitted diseases among men who have sex with men in 2010]**. *Shanghai Journal of Preventive Medicine* 2011, **23**(8):373-374.

70. Xu XY, Liu SY, Zhang JF: **[Analysis of AIDS sentinel surveillance in Huhhot in 2009]**. *Journal of Disease Monitor & Control* 2011, **5**(7):393-394, 390.

71. Zhang DD, Zhang Y, Li HL, Wang JX, Zhang S, Zhou WY, Wang CZ: **[Investigation of HIV, Syphilis and HCV Infection Situation and Sexual Behavioral Characteristics among MSM in Ningbo City]**. *Zhejiang Preventive Medicine* 2010, **22**(8):1-3.

72. Zhang H, Chen CG, Lin FH, Xu SY, Yao X, Lin H, Liu MY: **[Analysis of Comprehensive Surveillance Results of HIV/AIDS-related High-risk Groups in Fuzhou City in 2010]**. *Occupation and Health* 2011, **27**(21):2406-2409.

73. Zhang M, Wang XD, Yang Y: **[Prevalence oh HIV, anti-HCV, syphilis infection and AIDS knowledge among men who have sex with men (MSM) in Urumqi]**. *Chinese Journal of Public Health* 2009, **25**(9):1075-1076.

74. Zhang XY, wang C, Li XX, Zhang XX, Song YH, Li DL, Li SW, Ma LY, Shi W, Xu JQ *et al*: **[Study of HIV infection, co-infection with STDs and HCV and related changes in immunological indicators and viral loads among men who have sex with men in Beijing]**. *Chinese Journal of AIDS & STD* 2006, **12**(4):294-296, 320.

75. Zhang Y, Liu JW, Ni MJ, Dong YH: **[Laboratory Study on Detective Results of Male Homosexuality Population in Urumqi, Xinjiang]**. *Endemic Disease Bulletin* 2008, **23**(2):26-28.

76. Zhang ZK, Wen XQ, Chen W, Jiang W, Zhou Y: **[Survey on HIV/AIDS high-risk behaviors among men having sex with men in Guilin]**. *Disease Surveillance* 2010, **25**(3):213-215.

77. Zhao XH, Yang HW, He J, Jia SG, Yang H, Yao W: **[Surveillance of AIDS Related Knowledge Awareness, Risk Behaviors and Biology Among Men Having Sex With Men in Mianyang]**. *Journal of Preventive Medicine Information* 2010, **26**(11):876-878.

78. Zhou C, Ding XB, Feng L, Guo X, Han M, Xu S, Ouyang L, Ling H, Xu J: **[Study on the prevalence and associated factors of HIV and syphilis among 1166 men who have sex with men]**. *Modern Preventive Medicine* 2011, **38**(5):815-820.

79. Zhou CX, Pan ZP, Chen ZY, Guo HJ: **[Results analysis of AIDS sentinel surveillance in Zunyi City, 2010]**. *Jiangsu Journal of Preventive Medicine* 2011, **22**(3):11-13.

80. Zhou CX: **[Study on HIV infection among men who have sex with men in Zunyi city, Guizhou province]**. *Anhui Journal of Prevent Medicine* 2010, **16**(3):211-212.

81. Chen XX, Yu JP, Li M, Su QX: **[Survey on infection of HPV and HIV among men who have sex with men in Beijing]**. *International Journal of Virology* 2011, **18**(4):101-105.

82. Gao L, Zhou F, Li X, Yang Y, Ruan Y, Jin Q: **Anal HPV Infection in HIV-Positive Men Who Have Sex with Men from China**. *PLoS One* 2010, **5**(12):e15256.

83. Cai WD, Fen TJ, Tan JQ, Chen L, Shi XD, Chen PL, Jiang LZ, Tao XY: **[A Survery Of The Characteristics And STD/HIV Infection Of Homosexuality In Shenzhen]**. *Modern Preventive Medicine* 2005, **32**(4):328-330.

84. Feng Y, Wu Z, Detels R, Qin G, Liu L, Wang X, Wang J, Zhang L: **HIV/STD prevalence among men who have sex with men in Chengdu, China and associated risk factors for HIV infection**. *J Acquir Immune Defic Syndr* 2010, **53 Suppl 1**:S74-80.

85. Yin YP, Chen SC, Wang HC, Wei WH, Wang QQ, Liang GJ, Jiang N, Han Y, Chen XS, Wang B: **Prevalence and risk factors of HSV-2 infection and HSV-2/HIV coinfection in men who have sex with men in China: a multisite cross-sectional study**. *Sex Transm Dis* 2012, **39**(5):354-358.

86. Zheng M, Yao YM, Shen LM, Qin O, Zhou J: **[Survey of HIV/AIDS infection among men who have sex with men in Guiyang city of Guizhou province]**. *Guizhou Medical Journal* 2011, **35**(9):830-831.

87. Chen SH, Zhou J, Zhu JJ: **[Investigation of STI among some Men Who Have Sex with Men in Nanning City in 2006]**. *Preventive Medicine Tribune* 2007, **13**(9):772-774.
