# Supplementary material for: Disparities and Risks of Sexually Transmissible Infections among Men Who Have Sex with Men in China: A Meta-Analysis and Data Synthesis
Source: PLoS One. 2014 Feb 24;9(2):e89959. doi: 10.1371/journal.pone.0089959 (PMC3933676; doi:10.1371/journal.pone.0089959)
Supplement: Table S2 — Systematic review of 14 studies reporting the co-infection prevalence of sexually transmitted infections and/or viral hepatitis infections among HIV-positive men who have sex with men in China. (DOC) [file pone.0089959.s013.doc]

**Table S2**. Systematic review of 14 studies reporting the co-infection prevalence of sexually transmitted infections and/or viral hepatitis infections among HIV-positive men who have sex with men in China.

| **Study** | **Year** | **Province** | **Region^#^** | **Age** | **Age Range** | **Study Design^ϕ^** | **Recruitment method**^φ^ | **Sampling method** | **Co-infection** | | |
| --- | --- | --- | --- | --- | --- | --- | --- | --- | --- | --- | --- |
|  |  |  |  |  |  |  |  |  | **Number of HIV-positive MSM** | **Number of cases** | **Prevalence (%)** |
| ***a) Chlamydia*** | | | | | | | | | | | |
| Zhang X, 2007 | 2006 | Beijing | N | 26.1 | 18-55 | C-S | VCT | Pilot survey | 16 | 5 | 31.25 |
| ***b) Gonorrhoea*** | | | | | | | | | | | |
| Zhou J, 2008 | 2006 | Guizhou | SW | - | 15-49 | C-S | MSM venues, Internet advertisement, telephone hotline | - | 24 | 1 | 4.17 |
| ***b) HBV*** | | | | | | | | | | | |
| Guo H, 2009 | 2007 | Jiangsu | E | - | - | C-S | MSM venues | - | 25 | 4 | 16.00 |
| Wang WH, 2011 | - | Beijing | N | 29.8 | 20-57 | C-S | - | - | 61 | 10 | 16.39 |
| Zhang R, 2011 | 2006 | Zhejiang | E | 28.6 | 18-52 | C-S | MSM venues | Snowball | 41 | 13 | 31.71 |
| Zhou J, 2008 | 2006 | Guizhou | SW | - | 15-49 | C-S | MSM venues, Internet advertisement, telephone hotline | - | 24 | 1 | 4.17 |
| ***c) HCV*** | | | | | | | | | | | |
| Feng LG, 2010 | 2008 | Chongqing | SW | - | - | C-S | Internet; MSM venues | Snowball | 149 | 36 | 24.16 |
| Guo H, 2009 | 2007 | Jiangsu | E | - | - | C-S | MSM venues | - | 25 | 0 | 0 |
| Hu JF, 2011 | 2010 | Jiangxi | E | 22 | 16-45 | C-S | Community outreach, peer referral | - | 14 | 1 | 7.14 |
| Huang H, 2011 | 2008 | Anhui | E | 27.6 | 18-72 | C-S | MSM venues | Snowball | 12 | 0 | 0 |
| Sun ML, 2009 | 2008 | Liaoning | NE | - | 18-59 | C-S | MSM venues, VCT | Pilot survey | 17 | 1 | 5.88 |
| Wang T, 2010 | 2009 | Guangdong | E | 26 | 17-52 | C-S | VCT | - | 11 | 0 | 0 |
| Wang WH, 2011 | - | Beijing | N | 29.8 | 20-57 | C-S | - | - | 61 | 0 | 0 |
| Zhang R, 2011 | 2006 | Zhejiang | E | 28.6 | 18-52 | C-S | MSM venues | Snowball | 41 | 0 | 0 |
| Zhang X, 2007 | 2006 | Beijing | N | 26.1 | 18-55 | C-S | VCT | Pilot survey | 16 | 3 | 18.75 |
| Zhang XY, 2006 | 2005 | Beijing | N | - | - | C-S | - | - | 17 | 3 | 17.65 |
| ***d) HPV*** | | | | | | | | | | | |
| Chen XX, 2011 | 2010 | Beijing | N | - | - | C-S | NGOs | - | 29 | 28 | 96.55 |
| Gao L, 2012 | 2010 | Beijing and Tianjin | N | - | - | C-S | Internet advertisement, distributing flyers with study-related information at MSM venues | - | 50 | 48 | 96.00 |
| ***e) Herpes Simplex Virus Type 2*** | | | | | | | | | | | |
| Yin YP, 2012 | 2010 | Guangdong, Jiangsu | - | 30.14 | 18-66 | C-S | STD clinic, health centre, MSM venues | - | 139 | 47 | 33.81 |
| ***f) Ureaplasma urealyticum*** | | | | | | | | | | | |
| Zhang X, 2007 | 2006 | Beijing | N | 26.1 | 18-55 | C-S | VCT | Pilot survey | 16 | 3 | 18.75 |

^#^­Six Chinese traditional regions. E: East (Anhui, Fujian, Jiangsu, Jiangxi, Shandong, Shanghai, and Zhejiang); N: North (Beijing, Hebei, Inner Mongolia, Shanxi, and Tianjin); NE: Northeast (Heilongjiang, Jilin, and Liaoning; North China); NW: Northwest (Gansu, Ningxia, Qinghai, Shaanxi, and Xinjiang); SC: South Central (Guangdong, Guangxi, Hainan, Henan, Hubei, and Hunan); SW: Southwest (Chongqing, Guizhou, Sichuan, Tibet, and Yunnan).

^ϕ^C-S: Cross-sectional study;

^φ^ CDC: recruited by Chinese Center for Disease Control and Prevention; MSM venues: gay-oriented venues such as gay bars, saunas, bathhouse, and clubs; NGOs: recruited by non-governmental organisations. VCT: HIV voluntary counselling and testing sites;

**References:**

1. Zhang X, Wang C, Hengwei W, Li X, Li D, Ruan Y, Shao Y: **Risk factors of HIV infection and prevalence of co-infections among men who have sex with men in Beijing, China**. *AIDS* 2007, **21 Suppl 8**:S53-57.

2. Zhou J, Zhu JJ, Bin H, Zhang L, Yao M, Zhang W, Zhang W, Zhong JY, You QY, Gao L: **[A survey of HIV/STD, HBV and HCV infections and risk behaviors among MSM in two central districts of Guiyang city]**. *Chinese Journal of AIDS & STD* 2008, **14**(1):47-48, 51.

3. Guo H, Wei JF, Yang H, Huan X, Tsui SK, Zhang C: **Rapidly increasing prevalence of HIV and syphilis and HIV-1 subtype characterization among men who have sex with men in Jiangsu, China**. *Sex Transm Dis* 2009, **36**(2):120-125.

4. Wang WH, Ma Y, Jiang SL, Yang Y, Zhang XX, Shao YM, Zhang XY, Zhang FM: **[Investigation on HIV and hepatitis C co-infection among men who have sex with men in Beijing]**. *Chinese Journal of Public Health* 2011, **27**(5):624-625.

5. Zhang R, Shuai HQ, Shang XC: **[Investigation on the infection of HIV and Sex Transmitted Diseases in the man who have sex with man from 2006 to 2009 in Hangzhou]**. *Chinese Journal of Health Laboratory Technology* 2011, **21**(3):732-733.

6. Feng LG, Ding XB, Xu j, Ou YL, Xu SM, Zheng JQ, Guo XJ, Yang MF, Liu XP: **[Study on HIV, Syphilis and HCV Prevalence and Its Assoicated Factors among Internet MSM Comparison to Non-Internet MSM in Chongqing]**. *Journal of Tropical Medicine* 2010, **10**(1):78-82.

7. Hu JF, Wang YN, Li J: **[Study on HIV, syphilis and HCV infection and high risk behaviors among men who have sex with men in Donghu, Jiangxi Province]**. *World Health Digest* 2011, **8**(17):68-69.

8. Huang HY, Zhang XP, Li Y, Hu ZW, Li ZR: **[Study on the HIV, syphilis and hepatitis C infections and sexual behavior characteristics among men who have sex with men in Hefei]**. *Modern Preventive Medicine* 2011, **38**(10):1933-1935, 1938.

9. Sun ML, Li DJ, Jin W, Jiang J, Guan L: **[Investigation on the Infection of HIV, HCV, Syphilis and HBV among MSM in Dalian City in 2008]**. *Preventive Medicine Tribune* 2009, **15**(11):1074 - 1075.

10. Wang T, Lai XH, Li L, Chen CY, He BH: **[Survey on AIDS/STD Risk Behaviors and Prevalence Among Men Who Have Sex with Men in Zhongshan, Guangdong]**. *Practical Preventive Medicine* 2010, **17**(7):1261-1263.

11. Zhang XY, wang C, Li XX, Zhang XX, Song YH, Li DL, Li SW, Ma LY, Shi W, Xu JQ *et al*: **[Study of HIV infection, co-infection with STDs and HCV and related changes in immunological indicators and viral loads among men who have sex with men in Beijing]**. *Chinese Journal of AIDS & STD* 2006, **12**(4):294-296, 320.

12. Chen XX, Yu JP, Li M, Su QX: **[Survey on infection of HPV and HIV among men who have sex with men in Beijing]**. *International Journal of Virology* 2011, **18**(4):101-105.

13. Gao L, Zhou F, Li X, Yang Y, Ruan Y, Jin Q: **Anal HPV Infection in HIV-Positive Men Who Have Sex with Men from China**. *PLoS One* 2010, **5**(12):e15256.

14. Yin YP, Chen SC, Wang HC, Wei WH, Wang QQ, Liang GJ, Jiang N, Han Y, Chen XS, Wang B: **Prevalence and risk factors of HSV-2 infection and HSV-2/HIV coinfection in men who have sex with men in China: a multisite cross-sectional study**. *Sex Transm Dis* 2012, **39**(5):354-358.
