# Supplementary material for: Disparities and Risks of Sexually Transmissible Infections among Men Who Have Sex with Men in China: A Meta-Analysis and Data Synthesis
Source: PLoS One. 2014 Feb 24;9(2):e89959. doi: 10.1371/journal.pone.0089959 (PMC3933676; doi:10.1371/journal.pone.0089959)
Supplement: Table S3 — Publication bias and heterogeneity in subgroup meta-analyses. (DOC) [file pone.0089959.s014.doc]

**Table S3**. Publication bias and heterogeneity in subgroup meta-analyses

| **Study characteristics** | **Number of estimates (*n)*** | **Heterogeneity** | | | **Publication bias** |
| --- | --- | --- | --- | --- | --- |
|  |  | *Q*-value | *I*^2^ | *p-*value | Begg’s  (*p* values) |
| ***Chlamydia*** | 12 | 223.59 | 95.08 | <0.001** | 0.131 |
| *Urethral* | 8 | 50.15 | 86.04 | <0.001** | - |
| *Rectal* | 3 | 47.16 | 95.76 | <0.001** | - |
| ***Genital warts*** | 3 | 1.25 | 0.00 | 0.537 | 0.602 |
| ***Gonorrhoea*** | 14 | 51.04 | 74.53 | <0.001** | 0.154 |
| *Urethral* | 4 | 6.17 | 67.59 | 0.046* | - |
| *Rectal* | 3 | 3.18 | 5.75 | 0.364 | - |
| ***HBV*** | 35 | 165.97 | 79.51 | <0.001** | 0.081 |
| *East region* | 13 | 14.30 | 16.10 | 0.282 | - |
| *North region* | 8 | 18.97 | 63.10 | 0.008* | - |
| *Northeast region* | 4 | 4.58 | 34.44 | 0.206 | - |
| *South Central region* | 7 | 9.07 | 33.87 | 0.170 | - |
| *Southwest region* | 3 | 23.61 | 91.53 | <0.001** | - |
| *Year 2003* | 3 | 4.20 | 52.43 | 0.122 | - |
| *Year 2004* | 2 | 8.92 | 88.79 | 0.003* | - |
| *Year 2005* | 3 | 6.29 | 68.22 | 0.043* | - |
| *Year 2006* | 8 | 27.72 | 74.74 | <0.001** | - |
| *Year 2007* | 7 | 42.63 | 85.93 | <0.001** | - |
| *Year 2008* | 7 | 50.72 | 88.17 | <0.001** | - |
| *Year 2009* | 2 | 0.39 | 0.00 | 0.532 | - |
| *Year 2010* | 0 | - | - | - | - |
| *Year 2011* | 1 | 0.00 | 0.00 | 1.000 | - |
| ***HCV*** | 81 | 335.37 | 76.15 | <0.001** | 0.045 |
| *East region* | 23 | 28.30 | 22.25 | 0.166 | - |
| *North region* | 14 | 67.26 | 80.67 | <0.001** | - |
| *Northeast region* | 4 | 2.77 | 0.00 | 0.429 | - |
| *Northwest region* | 5 | 5.05 | 20.76 | 0.282 | - |
| *South Central region* | 22 | 126.90 | 83.45 | <0.001** | - |
| *Southwest region* | 12 | 42.21 | 73.94 | <0.001** | - |
| *Year 2003* | 3 | 2.32 | 13.89 | 0.313 | - |
| *Year 2004* | 2 | 0.01 | 0.00 | 0.930 | - |
| *Year 2005* | 4 | 25.84 | 88.39 | <0.001** | - |
| *Year 2006* | 9 | 21.79 | 63.29 | 0.005* | - |
| *Year 2007* | 11 | 4.04 | 0.00 | 0.946 | - |
| *Year 2008* | 23 | 91.01 | 75.83 | <0.001** | - |
| *Year 2009* | 15 | 93.90 | 85.09 | <0.001** | - |
| *Year 2010* | 10 | 6.16 | 0.00 | 0.724 | - |
| *Year 2011* | 1 | 0.00 | 0.00 | 1.000 | - |
| ***HPV*** | 2 | 6.08 | 83.56 | 0.014* | - |
| ***HSV-2*** | 6 | 126.81 | 96.06 | <0.001** | - |
| ***Ureaplasma urealyticum*** | 2 | 0.13 | 0.00 | 0.718 | - |

Note: Tests of publication bias and heterogeneity were not appropriate for meta-analyses with less than 3 studies. **p* < 0.05; ** *p* < 0.001.
